# One-pot three-component route for the synthesis of *S*-trifluoromethyl dithiocarbamates using Togni’s reagent

**DOI:** 10.3762/bjoc.13.247

**Published:** 2017-11-24

**Authors:** Azim Ziyaei Halimehjani, Martin Dračínský, Petr Beier

**Affiliations:** 1 Faculty of Chemistry, Kharazmi University, P. O. Box 15719-14911, 49 Mofateh St., Tehran, Iran; 2 The Institute of Organic Chemistry and Biochemistry of the Czech Academy of Sciences, Flemingovo namestí 2, 166 10 Prague 6, Czech Republic

**Keywords:** dithiocarbamates, electrophilic trifluoromethylation, Togni reagents

## Abstract

A one-pot three-component route for the synthesis of *S*-trifluoromethyl dithiocarbamates by the reaction of secondary amines, carbon disulfide and Togni’s reagent is described. The reactions proceed in moderate to good yields. A similar reaction using a primary aliphatic amine afforded the corresponding isothiocyanate in high yield. A variable temperature NMR study revealed a rotational barrier of 14.6, 18.8, and 15.9 kcal/mol for the C–N bond in the dithiocarbamate moiety of piperidine, pyrrolidine, and diethylamine adducts, respectively. In addition, the calculated barriers of rotation are in reasonable agreement with the experiments.

## Introduction

Dithiocarbamates are well known for their manifold applications as pesticides, fungicides and crop protection agents in agriculture [1–3], as intermediates in synthetic organic chemistry [4–12], radical chain transfer agents in RAFT polymerization [13], sulfur vulcanization agents in rubber manufacturing [14] and valuable pharmacophores in medicine [15–17]. Beside traditional methods, a recent synthesis of dithiocarbamates via a one-pot reaction of an amine, CS_2_ and an electrophile is of great interest due to its simplicity and environmental friendly procedure. Diverse electrophiles including alkyl halides [18], epoxides [19], alkenes [20–22], aldehydes [23], and alcohols [24] were applied for the synthesis of novel dithiocarbamates. Nevertheless, new synthetic methods towards dithiocarbamates are sought after and research in this area is still intense.

**Scheme 1:**
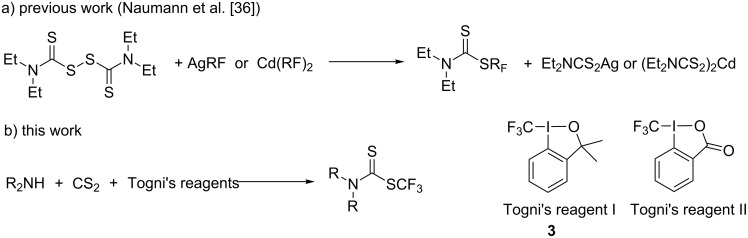
Synthetic routes for the preparation of trifluoromethyl dithiocarbamates.

The introduction of a trifluoromethyl group into organic compounds is a very productive strategy of modification of molecules for various applications in the fields of pharmaceuticals, agrochemicals, and materials sciences. The key properties such as metabolic and chemical stability, polarity, bioavailability, viscosity and lipophilicity can be altered in molecules containing the CF_3_ group in comparison with the nonfluorinated analogues. Numerous methods have been reported to introduce the trifluoromethyl group in organic structures including nucleophilic, electrophilic, radical and transition metal-mediated trifluoromethylations. Among the electrophilic trifluoromethylation methods, reagents based on hypervalent iodine (Togni's reagents I and II, Scheme 1b) have been used extensively in trifluoromethylations of S-, P-, O-, and C-nucleophilic functionalities [25–32]. Although reports exist on the synthesis of fluorinated dithiocarbamates [33–35], the direct trifluoromethylation of dithiocarbamates has not been described. In 2001 Naumann and co-workers [36] have published a reaction of tetraethylthiuram disulfide with perfluoroorganosilver and perfluoroorganocadmium reagents proceeding most probably by a radical mechanism (Scheme 1a). This method suffers from several drawbacks such as extra reaction steps for the preparation of the thiuram disulfide and the use of expensive and environmentally problematic heavy metals. These observations prompted us to investigate the reaction of in situ prepared dithiocarbamic acid with Togni's reagents as a new route to *S*-trifluoromethyl dithiocarbamates (Scheme 1b).

## Results and Discussion

We started our investigation with a one-pot, three-component reaction of piperidine, CS_2_, and a cyclic hypervalent iodane (Togni's reagent I) as a model reaction to find the optimal reaction conditions for the preparation of *S*-trifluoromethyl dithiocarbamates (Table 1). In the first run, we observed that the reaction of piperidine (1 equiv) with CS_2_ (2 equiv) in THF for 10 min followed by the addition of Togni's reagent I (1 equiv) and stirring for 2 hours at room temperature afforded the corresponding trifluoromethyl dithiocarbamate **4a** in 25% isolated yield and the corresponding thiuram disulfide **5a** (ratio of **4a**:**5a** 1.8:1) (Table 1, entry 1). Performing the same reaction at −78 °C and slow heating to ambient temperature over 2 hours increased the yield of **4a** to 35% with an increase in the **4a**:**5a** ratio to 2.4:1 (Table 1, entry 2). Using excess of both the amine (2 equiv) and CS_2_ (3 equiv) a higher yield of **4a** was obtained, albeit the product ratio decreased (Table 1, entry 3). By further varying the equivalents of amines and CS_2_, we found that when using 1.5 equivalents of piperidine and CS_2_ and 1 equivalent of **3** in THF at −78 °C for 1 hour, the yield was improved to 45% with a product ratio of 2.5:1 (Table 1, entry 4). Using excess of **2** and **3** with piperidine as the limiting reagent led to significant yield reduction (Table 1, entry 5). The use of chloroform, dichloromethane, ethanol or water as solvents afforded the product, albeit in unsatisfactory yield (interestingly, in aqueous KOH the **4a**:**5a** ratio was the highest observed, Table 1, entries 6–10). In addition, using Togni's reagent II in THF gave low yields of **4a** (Table 1, entry 11). Using Et_3_N as base in THF also afforded unsatisfactory results (Table 1, entry 12). Although with the potassium salt of piperidine dithiocarbamate in THF, water or DMF the product ratio increased to 5:1 (Table 1, entries 13–15), the yield of **4a** remained low compared to our one-pot three-component reaction in THF. In summary, stirring piperidine (1.5 equiv) and CS_2_ (1.5 equiv) in THF at room temperature for 10 min, followed by cooling to −78 °C, addition of **3** (1 equiv) and additional stirring for one hour at −78 °C were considered as optimal reaction conditions for further derivatization.

**Table 1:** Optimization of the reaction conditions.

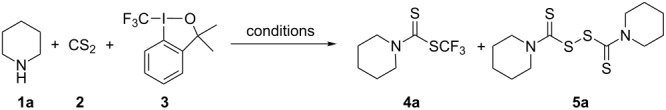								

Entry	**1a** (equiv)	**2** (equiv)	**3** (equiv)	Solvent	*T* (°C)	Time (h)	Yield **4a** (%)^a^	**4a**:**5a**^b^

1	1	2	1	THF	rt	2	25	1.8:1
2	1	2	1	THF	−78 to rt	2	35	2.4:1
3	2	3	1	THF	−78	1	40	1.75:1
4	1.5	1.5	1	THF	−78	1	**45**	**2.5:1**
5	1	2	1.2	THF	−78	1	22	1.6:1
6	1.5	2	1	CHCl_3_	0-5	1	17	1:1.4
7	1	2	1	CHCl_3_	rt	1	15	1.6:1
8	1.5	1.5	1	CH_2_Cl_2_	−78	2	13	1.1:1
9	1.2	1.2	1	EtOH	−78	2	15	1.7:1
10	1.5	1.5	1	H_2_O	0 to rt	1	30	6:1^c^
11	1.5	1.5	1	THF	−78	1	10	1:1.8^d^
12	1.5	1.5	1	THF	−78 to rt	2	10	1:1^e^
13		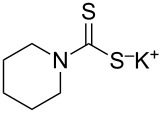 (1 equiv)	1	THF	−78 to rt	1.5	10	4:1
14		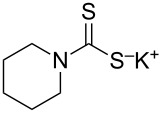 (1.5 equiv)	1	H_2_O	0 to rt	2	25	5:1
15		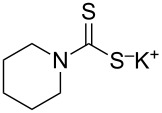 (1 equiv)	1	DMF	−55 to rt	1.5	12	1:1

^a^Isolated yield of **4a**. ^b^The ratio of **4a**:**5a** was determined by ^1^H NMR spectroscopy of the crude reaction mixture. ^c^KOH (1.5 equiv) was used. ^d^Togni's reagent II was used instead of Togni's reagent I (**3**). ^e^Et_3_N (1.5 equiv) was used.

**Scheme 2:**
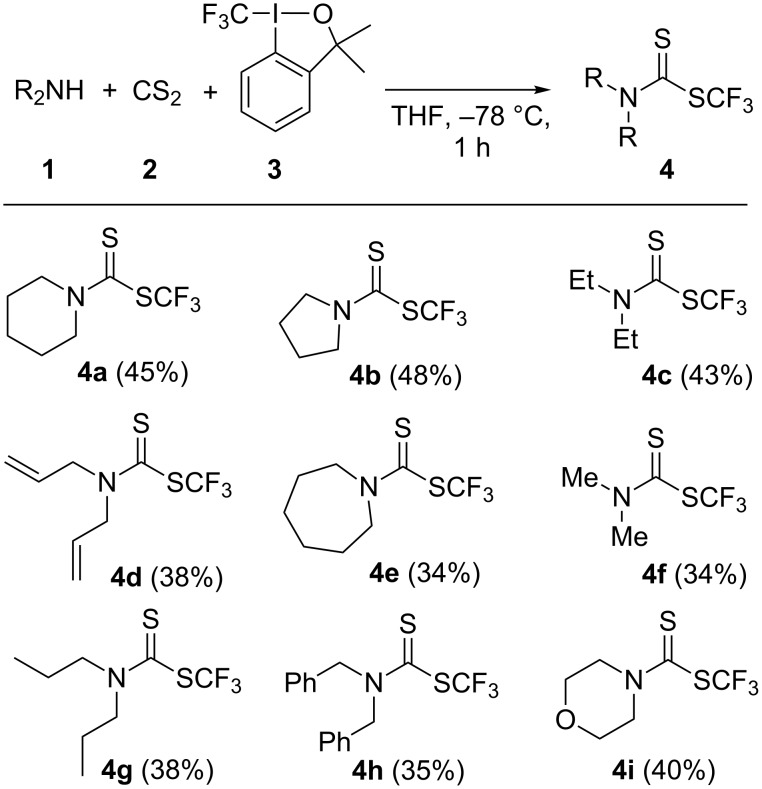
Synthesis of *S*-trifluoromethyl dithiocarbamates. Isolated yields are given in parentheses.

In order to explore the scope of the reaction under the optimized reaction conditions, various commercially available secondary amines were investigated and moderate to good yields of products **4** were obtained (Scheme 2). The products were isolated from the reaction mixture by extraction with CH_2_Cl_2_ and purification was carried out by column chromatography on silica gel using CH_2_Cl_2_/*n*-pentane (1:9) as eluent. The structure of products was confirmed using IR, ^1^H NMR, ^13^C NMR, ^19^F NMR and HRMS analysis. The dithiocarbamate moiety in *S*-trifluoromethyl dithiocarbamates appeared at 180–185 ppm in the ^13^C NMR spectra, while this group usually can be found at 190–200 ppm for *S*-alkyl dithiocarbamates [4–12]. Also the carbon of the CF_3_ group was observed at around 128 ppm as quartet with a coupling constant of ≈308 Hz. In addition, a singlet at −40 ppm in the ^19^F NMR spectra was assigned to the CF_3_ group.

**Scheme 3:**
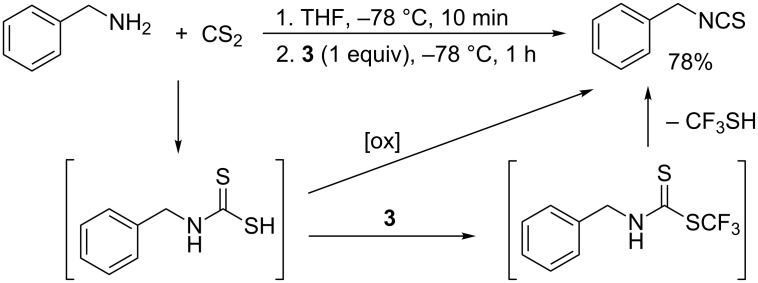
Formation of benzyl isothiocyanate in a reaction with benzylamine.

The one-pot reaction of benzylamine with CS_2_ and Togni's reagent I under optimal reaction conditions was also investigated. Surprisingly, we observed that the corresponding benzyl isothiocyanate was obtained in high yield. This may be attributed to the low stability of the corresponding *S*-trifluoromethyl benzyldithiocarbamate. Alternatively, the iodane **3** can act as an oxidant towards the intermediate benzyl dithiocarbamic acid rather than an electrophilic trifluoromethylating reagent (Scheme 3). A similar behavior was recently reported by Schoenebeck [37] who showed that isothiocyanates are formed in reactions of primary amines with the (Me_4_N)SCF_3_ salt (through a different mechanism).

Interesting differences in the NMR spectra of compounds **4a**–**i** were observed. The nitrogen–carbon bond in the dithiocarbamate group [N–C(S)S] has a bond order higher than one leading to nonequivalence of the two alkyl substituents attached to the nitrogen atom. However, the rotational barrier of the N–C bond differs significantly for individual compounds and ^1^H NMR spectra exhibit features of slow to intermediate chemical exchange with substantial signal broadening in some cases. Therefore, we performed a variable temperature NMR study to determine rotational barriers of compounds **4a**–**c**, and the experimental data are compared to and rationalized by DFT calculations.

**Figure 1:**
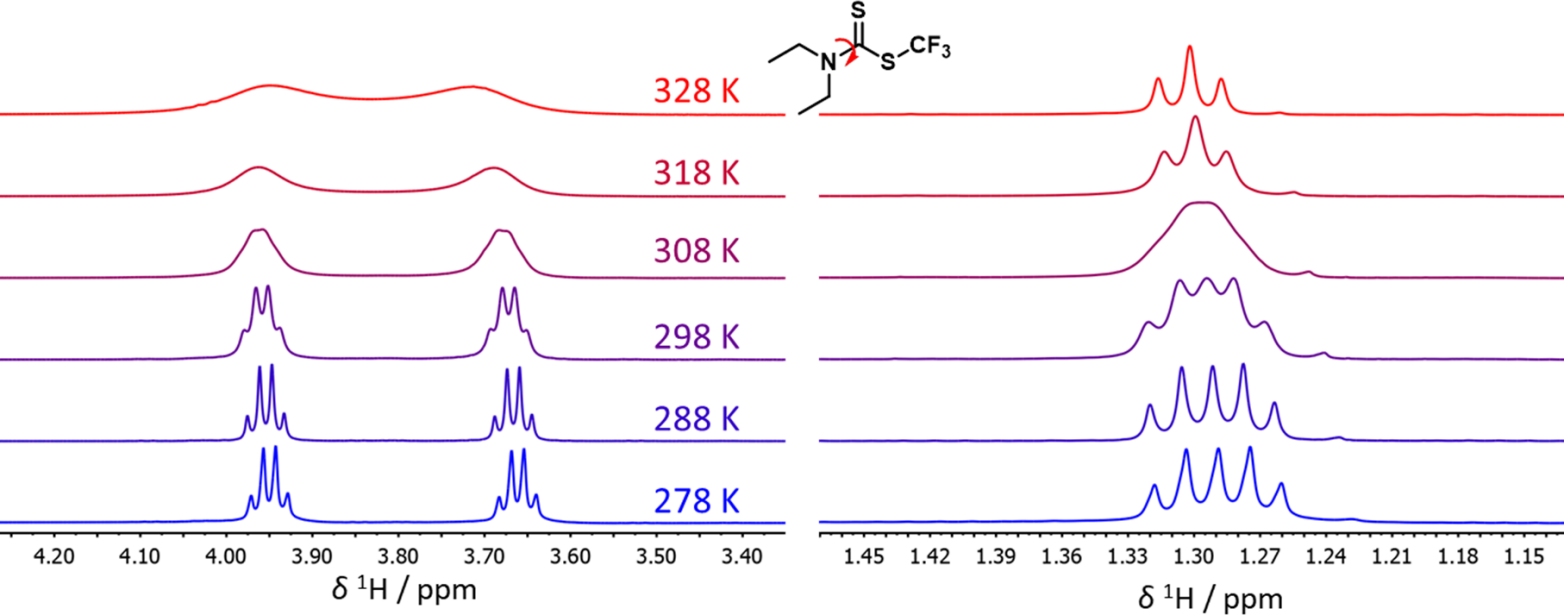
Variable temperature ^1^H NMR spectra of compound **4c** (CH_2_ region on the left and CH_3_ region on the right); the sample was dissolved in CDCl_3_.

**Figure 2:**
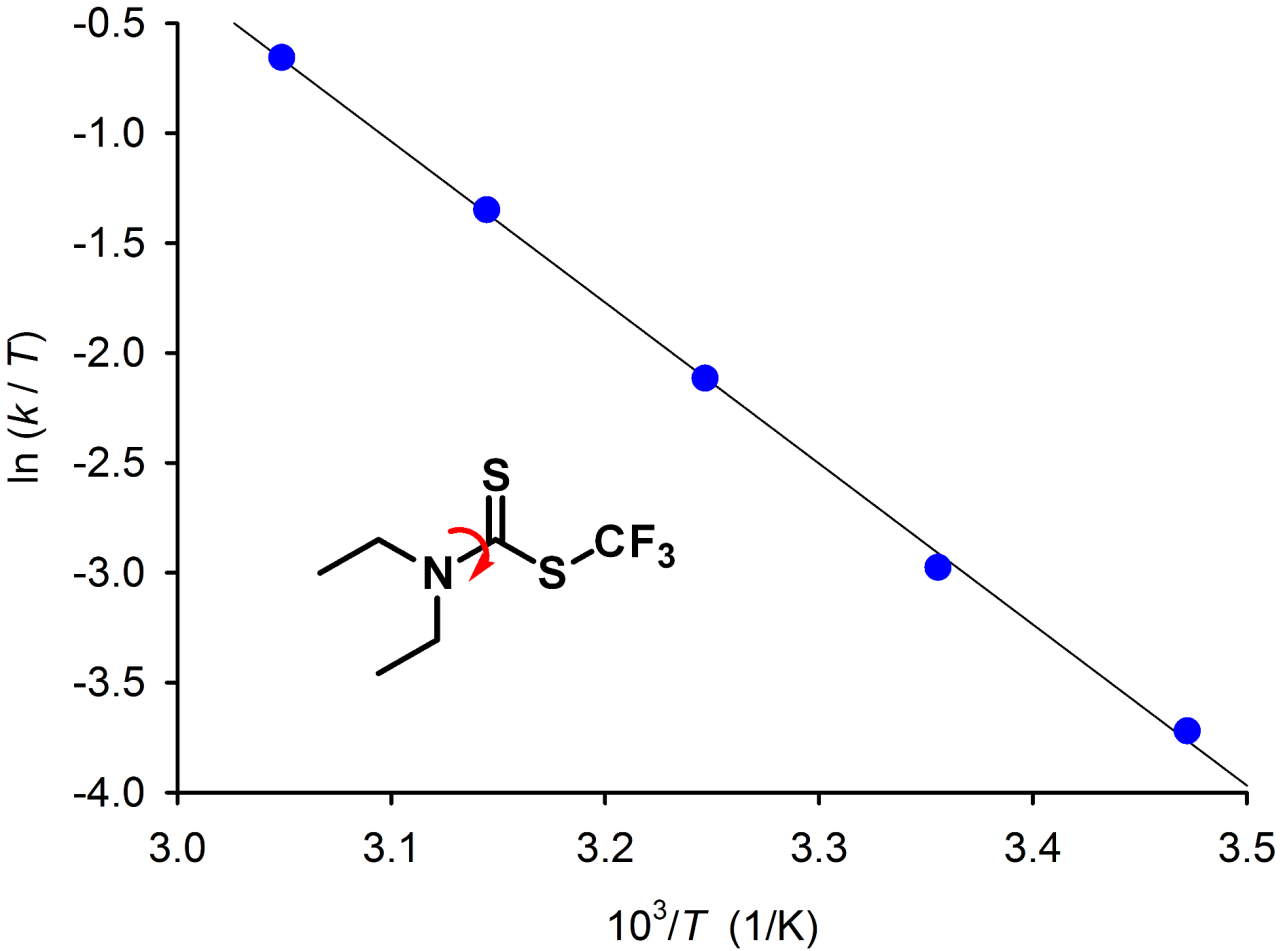
The Eyring plot obtained for the rotation around the N–C bond in compound **4c**.

Figure 1 depicts variable temperature ^1^H NMR spectra of compound **4c**. The coalescence of the methyl signals can be observed at 308 K, whereas the coalescence of the CH_2_ signals would require an even higher temperature than 328 K. Complete lineshape analysis approach (dynamic NMR, dNMR) provided the rates of rotation around the N–C bond at all temperatures and the Eyring plot (Figure 2) allowed to determine the enthalpy and entropy of activation. The entropy of activation is small (−4.2 cal/K) and the free energy of activation (15.8 kcal/mol at 300 K) is dominated by the enthalpic term (14.6 kcal/mol). The rate of rotation *k* at coalescence temperature can also be determined by applying the equation *k* = 2.22Δν, where Δν is the difference between resonance frequencies of the exchanging signals at slow exchange regime (at low temperature). The rotational barrier for compound **4c** determined using this approach (15.9 kcal/mol) is almost identical to that obtained by the dNMR approach and is higher compared to the corresponding nonfluorinated analogue [35,38–39]. The rotational barriers obtained for compounds **4a**–**c** are summarized in Table 2.

**Table 2:** Experimental and calculated (B3LYP/6-31+g**) rotational barriers (kcal/mol) in compounds **4a**–**c**.

Compound	Δ*G*^#^_exp_	Δ*E*^#^_calc_

**4a**	14.6	14.6
**4b**	18.8	16.3
**4c**	15.9	15.0

The structures of compounds **4a**–**c** was optimized using DFT methods (B3LYP/6-31+g**). In all three cases, the carbon atoms attached to the nitrogen and the N–C(S)–S–C atoms of the dithiocarbamate group are almost in one plane and the torsion angle S=C–S–CF_3_ is close to zero in the optimized structures. In the transition state corresponding to the rotation around the N–C bond, the plane with CH_2_ carbon atoms attached to the nitrogen and the nitrogen atom is almost perpendicular to the N–C(S)–S–C plane. The calculated barriers of rotation are in reasonable agreement with experimental data (Table 2).

**Figure 3:**
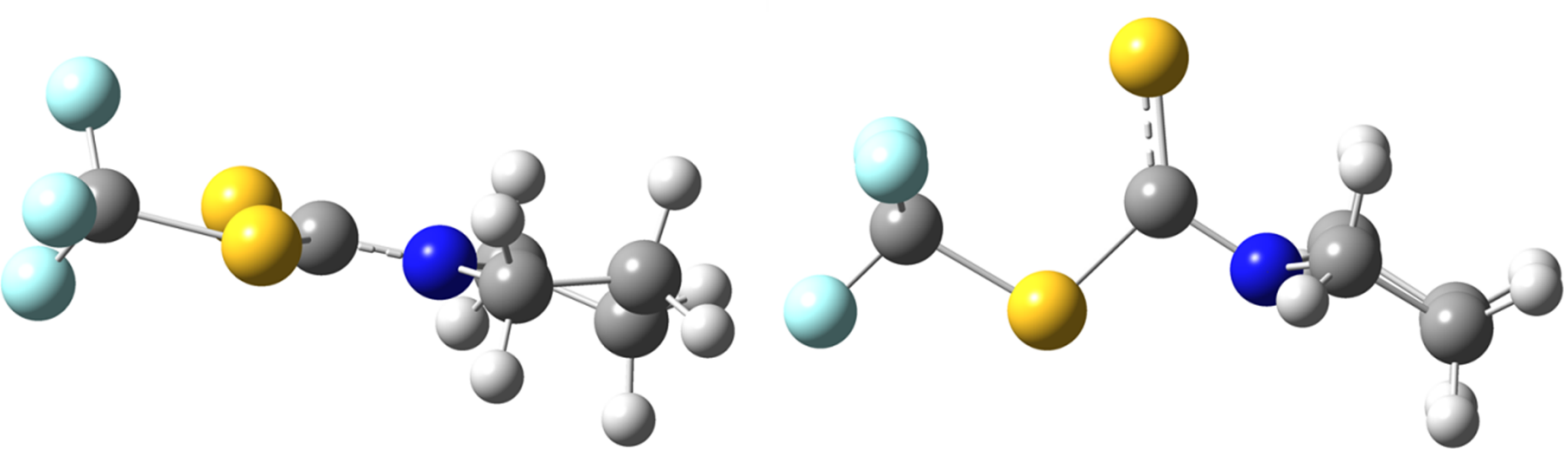
The optimized structure of compound **4b** (left) and the transition state structure for the rotation around the N–C bond (right).

The rotational barrier in compound **4b** is both experimentally and computationally higher than in the other two compounds (**4a** and **4c**). This can be explained by the conformational strain in the five-membered ring in the transition state. In the ground state, the conformation of the pyrrolidine ring is _4_T^3^ with limited steric interactions between adjacent CH_2_ hydrogen atoms (Figure 3). On the other hand, a ^1^E conformation is found in the transition state structure. Hydrogen atoms are close to unfavorable syn-periplanar arrangement in this conformation, which leads to an increased energy demand for the pyrrolidine rotation.

## Conclusion

In conclusion, we have reported a novel multicomponent reaction for the synthesis of *S*-trifluoromethyl dithiocarbamates from secondary amines, CS_2_ and Togni's reagent in moderate to good yields. Under similar conditions, a primary amine afforded the corresponding isothiocyanate in excellent yield. The presence of dithiocarbamate and trifluoromethyl groups in a single structure generates a new family of compounds with potential application as agrochemicals or in drug design. A variable temperature NMR study allowed the determination of rotational barriers of 14.6, 18.8, and 15.9 kcal/mol for the C–N bond in the dithiocarbamate moiety of piperidine, pyrrolidine and diethylamine adducts, respectively. The results revealed that the rotational barrier in fluorinated dithiocarbamates is slightly higher than in the nonfluorinated analogue [35,38–39]. This may be attributed to a higher electron affinity of the trifluoromethyl group and an increased double bond character of the C–N bond.

## Supporting Information

File 1 Experimantal procedures and characterization data of all products, copies of ^1^H, ^13^C, and ^19^F NMR spectra of all compounds.
