# Supplementary material for: One-pot three-component route for the synthesis of S-trifluoromethyl dithiocarbamates using Togni’s reagent
Source: Beilstein J Org Chem. 2017 Nov 24;13:2502–8. doi: 10.3762/bjoc.13.247 (PMC5704766; doi:10.3762/bjoc.13.247)
Supplement: File 1 — Experimantal procedures and characterization data of all products, copies of 1H, 13C, and 19F NMR spectra of all compounds. [file Beilstein_J_Org_Chem-13-2502-s001.pdf]

Supporting Information

for

One-pot three-component route for the synthesis of *S*-trifluoromethyl dithiocarbamates using Togni's reagent

Azim Ziyaei Halimehjani[*1], **Martin Dračínský**[2] and Petr Beier[*2]

Address: [1]Faculty of Chemistry, Kharazmi University, P. O. Box 15719-14911, 49 Mofateh St., Tehran, Iran and [2]The Institute of Organic Chemistry and Biochemistry of the Czech Academy of Sciences, Flemingovo namestí 2, 166 10 Prague 6, Czech Republic.

Email: Azim Ziyaei Halimehjani - ziyaei@khu.ac.ir; Petr Beier - beier@uochb.cas.cz

*Corresponding author

Experimantal procedures and characterization data of all products, copies of ^{1}H, ^{13}C, and ^{19}F NMR spectra of all compounds

Contents **page**

1. General S1
2. Experimental S2
3. Copies of NMR spectra S5
4. Computational (general information) S34
5. References S34

1. General

IR spectra were recorded on an FTIR instrument using a film technique and wave numbers are reported in cm^{-1}. NMR spectra were recorded on 400 or 500 MHz instruments at 300 K. In the case of **4a** and **4c**, the ^{13}C NMR spectra were recorded at 278 K. The chemical shifts (δ) are reported in parts per million (ppm) and coupling constants (*J*) are given in Hertz. ^{13}C and ^{19}F NMR spectra were proton decoupled. The chemical shifts are reported in ppm relative to Me_4Si (0 ppm for ^{1}H NMR in $CDCl_3$), residual $CHCl_3$ (7.26 ppm for ^{1}H NMR), $CDCl_3$ (77.16 ppm for ^{13}C NMR), and internal $CFCl_3$ (0 ppm for ^{19}F NMR). High-resolution mass spectra (HRMS) were recorded on an Agilent 7890A gas chromatograph coupled with a Waters GCT Premier orthogonal acceleration time-of-flight detector using electron impact (EI) ionizations.

2. Experimental

General Procedure for the synthesis of S-trifluoromethyl dithiocarbamates 4a–i: In a Schlenk flask under Ar or N_2 atmosphere, dry THF (4 mL), amine (1.5 mmol, 1.5 equiv) and CS_2 (1.5 mmol, 0.09 mL, 1.5 equiv) were added respectively. After stirring for 10 min at room temperature, the reaction mixture was cooled to −78 °C, and a solution of Togni reagent I (1 mmol, 0.33 g, 1 equiv; solution in 1 mL of THF) was added. The mixture was further stirred for 1 h at −78 °C. Water (5 mL) was added and the product was extracted into CH_2Cl_2 (3 × 10 mL). The organic extracts were combined, washed with water (2 × 10 mL), dried with anhydrous $MgSO_4$, and evaporated to give the crude products. Chromatography on silica gel, elution with CH_2Cl_2/*n*-pentane (1:9) afforded pure products.

Procedure for the synthesis of benzylisothiocyanate using Togni reagent I:

In a Schlenk tube under N_2 atmosphere, dry THF (4 mL), benzylamine (1.5 mmol, 1.5 equiv) and CS_2 (1.5 mmol, 0.09 mL, 1.5 equiv) were added respectively. After stirring for 10 min at room temperature, the reaction mixture was cooled to −78 °C, and a solution of Togni reagent I (1 mmol, 0.33 g, 1 equiv; solution in 1 mL of THF) was added. The mixture was further stirred for 1 h at −78 °C. Solvent was evaporated under reduced pressure to give the crude product. Chromatography on silica gel, elution with CH_2Cl_2/*n*-pentane (1:9) afforded pure benzylisothiocyanate in 78% isolated yield.

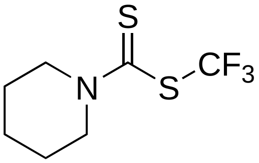

*Trifluoromethyl piperidine-1-carbodithioate (**4a**);* Yield: 103 mg (45%); yellow oil; FTIR (film) 2944 m, 2859 m, 1478 s, 1457 m, 1430 s, 1360 w, 1353 w, 1281 m, 1247 s, 1225 m, 1165 s, 1156 s, 1113 s, 1092 vs, 971 m, 852 m, 761 m; ^1H NMR (400 MHz, $CDCl_3$) δ 4.24 (br s, 2H), 3.85 (br s, 2H), 1.76–1.73 (m, 6H); ^{13}C NMR (126 MHz, $CDCl_3$) δ 181.2, 128.7 (q, J = 308.2 Hz, CF_3), 53.6, 51.6, 26.4, 25.2, 24.0; ^{19}F NMR (376 MHz, $CDCl_3$) δ -40.66 (s); HRMS calcd for $C_7H_{10}NS_2F_3$ $[M]^+$, 229.0207; Found 229.0206.

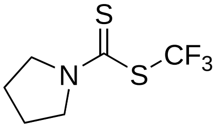

*Trifluoromethyl pyrrolidine-1-carbodithioate (**4b**);* Yield: 103 mg (48%); Cream solid; mp 59-62 °C; FTIR (film) 2989 w, 2957 w, 2926 w, 2902 w, 2879 w, 2855 w, 1481 m, 1473 m, 1451 m, 1444 m, 1332 w, 1255 m, 1190 s, 1179 s, 1167 vs, 1099 m, 956 m, 910 w, 762 m; ^1H NMR (401 MHz, $CDCl_3$) δ 3.91 (t, J = 7.0 Hz, 2H), 3.66 (t, J = 6.8 Hz, 2H), 2.21–2.09 (m, 2H), 2.09–1.96 (m, 2H); ^{13}C NMR (126 MHz, $CDCl_3$) δ 179.20, 128.5 (q, J = 310 Hz, CF_3), 54.6, 51.9, 26.3, 24.2; ^{19}F NMR (377 MHz, $CDCl_3$) δ -40.94 (s); HRMS calcd for $C_6H_8NS_2F_3$ $[M]^+$, 215.005; Found 215.0049.

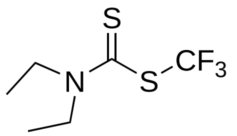

*Trifluoromethyl diethylcarbamodithioate (**4c**);* Yield: 93 mg (43%); Yellow oil; FTIR (film) 2981 m, 2937 w, 2876 w, 1492 s, 1463 m, 1457 m, 1444 m, 1420 s, 1384 m, 1273 s, 1208 s, 1158 s, 1100 s, 977 m, 761 m; ^{1}H NMR (400 MHz, $CDCl_3$) δ 3.98 (q, J = 7.0 Hz, 2H), 3.71 (q, J = 7.1 Hz, 2H), 1.30–1.35 (m, 6H); ^{13}C NMR (126 MHz, $CDCl_3$) δ 181.6, 128.7 (q, J = 307.4 Hz), 48.8, 48.5, 13.1, 11.2; ^{19}F NMR (376 MHz, $CDCl_3$) δ -40.97 (s); HRMS calcd for $C_6H_{10}NS_2F_3$ $[M]^+$, 217.0207; Found 217.0204.

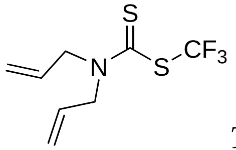

*Trifluoromethyl diallylcarbamodithioate (**4d**);* Yield: 92 mg (38%); Yellow oil; FTIR (film) 3087 w, 3016 w, 2987 vw, 2925 w, 2855 vw, 1476 m, 1434 m, 1403 s, 1277 m, 1236 s, 1171 s, 1156 s, 1099 vs, 992 m, 961 w, 762 m; ^{1}H NMR (400 MHz, $CDCl_3$) δ 5.89–5.79 (m, 2H), 5.36–5.23 (m, 4H), 4.60 (br s, 2H), 4.29 (br s, 2H); ^{13}C NMR (101 MHz, $CDCl_3$) δ 183.8, 129.8 (2C), 128.9 (q, J = 308.6 Hz), 119.4, 119.3, 55.8, 55.2; ^{19}F NMR (376 MHz, $CDCl_3$) δ -41.12 (s); HRMS calcd for $C_8H_{10}NS_2F_3$ $[M]^+$, 241.0207; Found 241.0204.

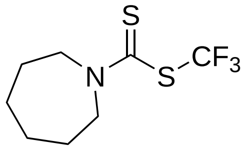

*Trifluoromethyl azepane-1-carbodithioate (**4e**);* Yield 83 mg (34%); Yellow oil; FTIR (film) 2934 m, 2859 m, 1494 s, 1449 m, 1440 m, 1418 s, 1367 m, 1354 m, 1274 s, 1201 s, 1170 s, 1150 s, 1100 vs, 1092 vs, 762 m; ^{1}H NMR (400 MHz, $CDCl_3$) δ 4.13 (t, J = 6.1 Hz, 2H), 3.84 (t, J = 6.1 Hz, 2H), 1.93–1.84 (m, 4H), 1.64–1.61 (m, 4H); ^{13}C NMR (101 MHz, $CDCl_3$) δ 182.3, 129.0 (q, J = 309.2 Hz, CF_3), 54.8, 54.5, 27.9, 26.4, 26.3, 25.6; ^{19}F NMR (376 MHz, $CDCl_3$) δ -40.94 (s); HRMS calcd for $C_8H_{12}NS_2F_3$ $[M]^+$, 243.0363; Found 243.0367.

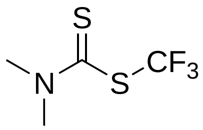

*Trifluoromethyl dimethylcarbamodithioate (**4f**);* Yield: 64 mg (34%); Yellow oil; FTIR (film) 2926 w, 1503 m, 1378 s, 1247 m, 1155 s, 1099 vs, 976 m, 762 w; ^{1}H NMR (400 MHz, $CDCl_3$) δ 3.50 (s, 3H), 3.41 (s, 3H); ^{13}C NMR (101 MHz, $CDCl_3$) δ 183.1, 128.8 (q, J = 308.3 Hz, CF_3), 44.5, 43.0; ^{19}F NMR (376 MHz, $CDCl_3$) δ -41.52 (s); HRMS calcd for $C_4H_6NS_2F_3$ $[M]^+$, 188.9894; Found 188.9895.

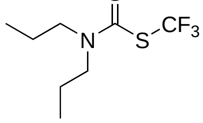

*Trifluoromethyl dipropylcarbamodithioate (**4g**);* Yield: 93 mg (38%); Yellow oil; FTIR (film) 2969 m, 2936 m, 2878 m, 1486 s, 1467 m, 1451 m, 1440 m, 1415 m, 1383 w, 1242 s, 1197 s, 1159 s, 1099 vs, 1084s, 987 m, 762 m; ^{1}H NMR (400 MHz, $CDCl_3$) δ 3.86 (t, J = 8.0 Hz, 2H), 3.59 (t, J = 7.9 Hz, 2H), 1.82–1.74 (m, 4H), 0.98 (m, 6H); ^{13}C NMR (101 MHz, $CDCl_3$)

δ 182.0, 129.0 (q, J = 308.1 Hz, CF_3), 56.0, 55.9, 21.5, 19.4, 11.1, 11.0; ^{19}F NMR (376 MHz, $CDCl_3$) δ -40.97 (s); HRMS calcd for $C_8H_{14}NS_2F_3$ $[M]^+$, 245.0520; Found 245.0526.

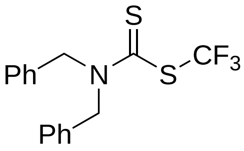

*Trifluoromethyl dibenzylcarbamodithioate (**4h**);* Yield 120 mg (35%); Yellow oil; FTIR (film) 3088 w, 3065 w, 3032 w, 3008 w, 2926 w, 2856 vw, 1496 s, 1472 s, 1453 s, 1438 m, 1413 s, 1222 s, 1167 s, 1095 vs, 1030 m, 1002 m, 961 m, 761 m, 697 s; ^{1}H NMR (400 MHz, $CDCl_3$) δ 7.45–7.24 (m, 10H), 5.29 (s, 2H), 4.88 (s, 2H); ^{13}C NMR (101 MHz, $CDCl_3$) δ 185.2, 134.6, 133.7, 129.2, 129.0, 128.4, 128.1, 126.9, 126.8, 125.9 (q, J = 308.6 Hz, CF_3), 55.7, 55.3; ^{19}F NMR (376 MHz, $CDCl_3$) δ -40.94 (s); HRMS calcd for $C_{16}H_{14}NS_2F_3$ $[M]^+$, 341.0520; Found 341.0526.

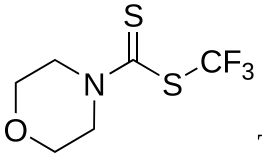

Trifluoromethyl morpholine-4-carbodithioate (**4i**); Yield 92 mg (40%); Yellow oil; FTIR (film) 2972 m, 2925 m, 2859 m, 1466 s, 1423 s, 1386 m, 1364 m, 1269 s, 1236 s, 1172 s, 1152 s, 1113 vs, 1096 vs, 988 s, 866 m, 761 s; ^{1}H NMR (400 MHz, $CDCl_3$) δ 4.09 (br s, 4H), 3.80–3.78 (m, 4H); ^{13}C NMR (101 MHz, $CDCl_3$) δ 182.5, 128.9 (q, J = 308.6 Hz, CF_3), 66.1 (2C), 51.8, 50.6; ^{19}F NMR (376 MHz, $CDCl_3$) δ -40.45 (s); HRMS calcd for $C_6H_8NOS_2F_3$ $[M]^+$, 230.9999; Found 231.0002.

3. Copies of NMR spectra

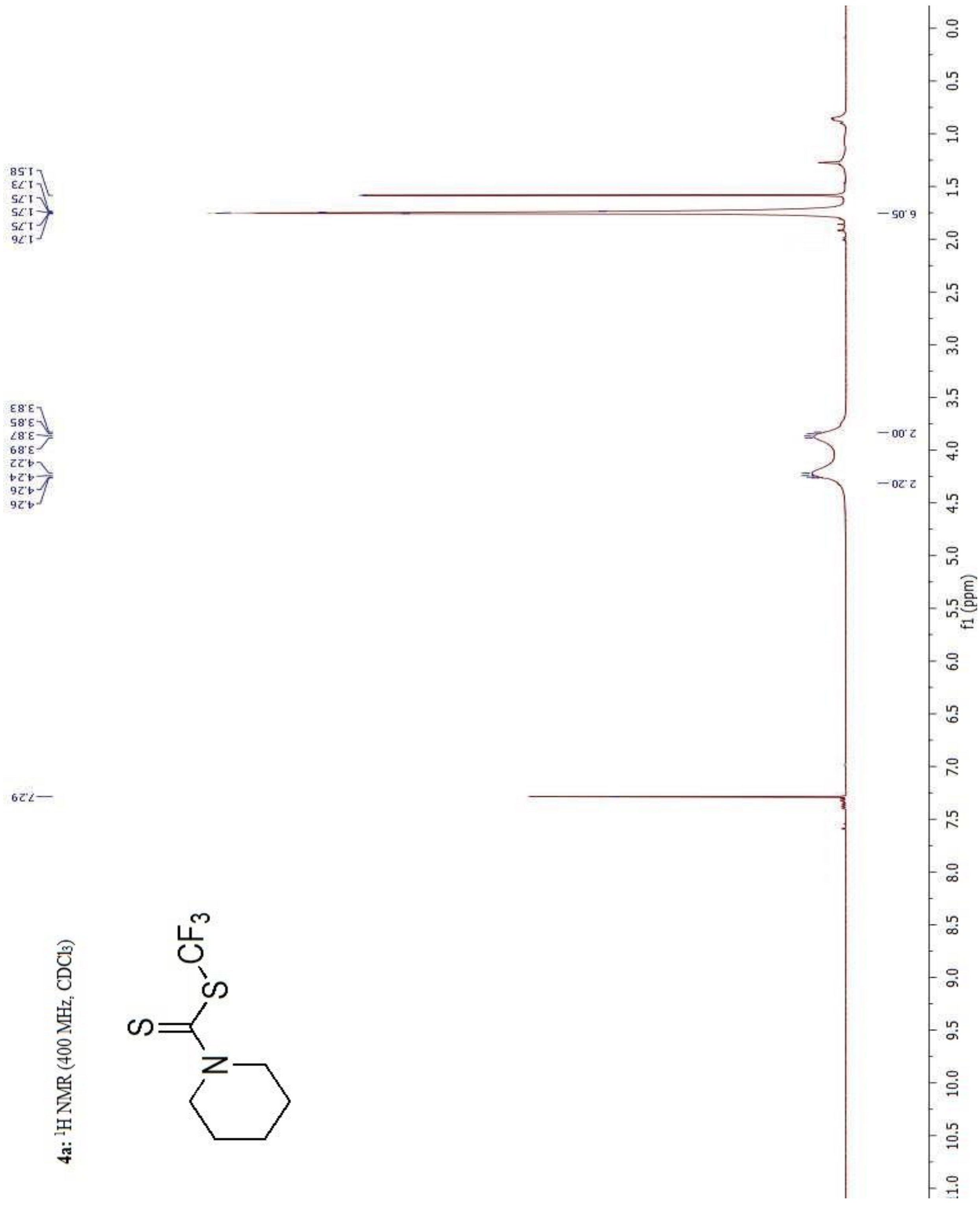

4a: ^{13}C NMR (126 MHz, $CDCl_3$, ^{13}C NMR is measured at 278 K)

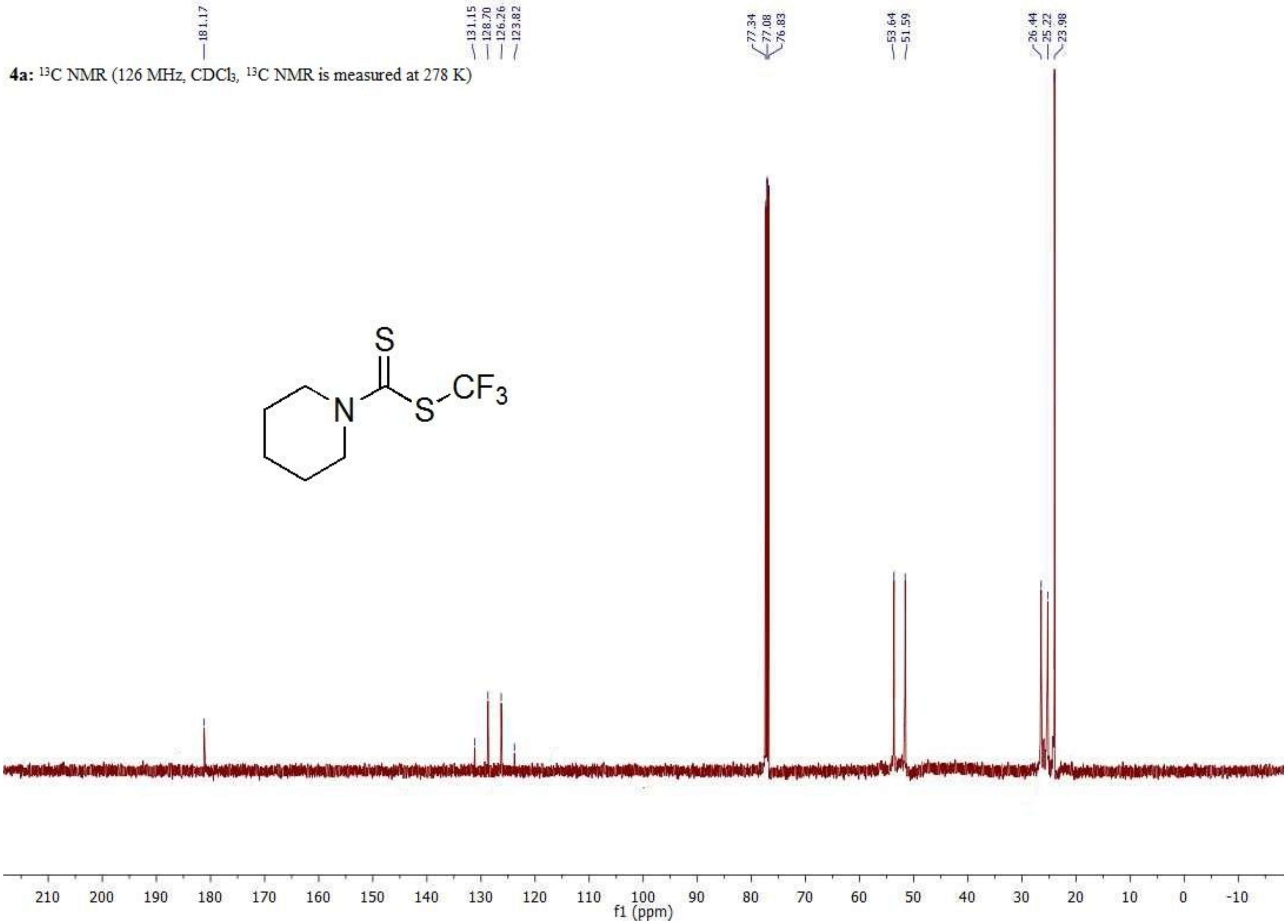

4a: ^{19}F NMR (376 MHz, $CDCl_3$)

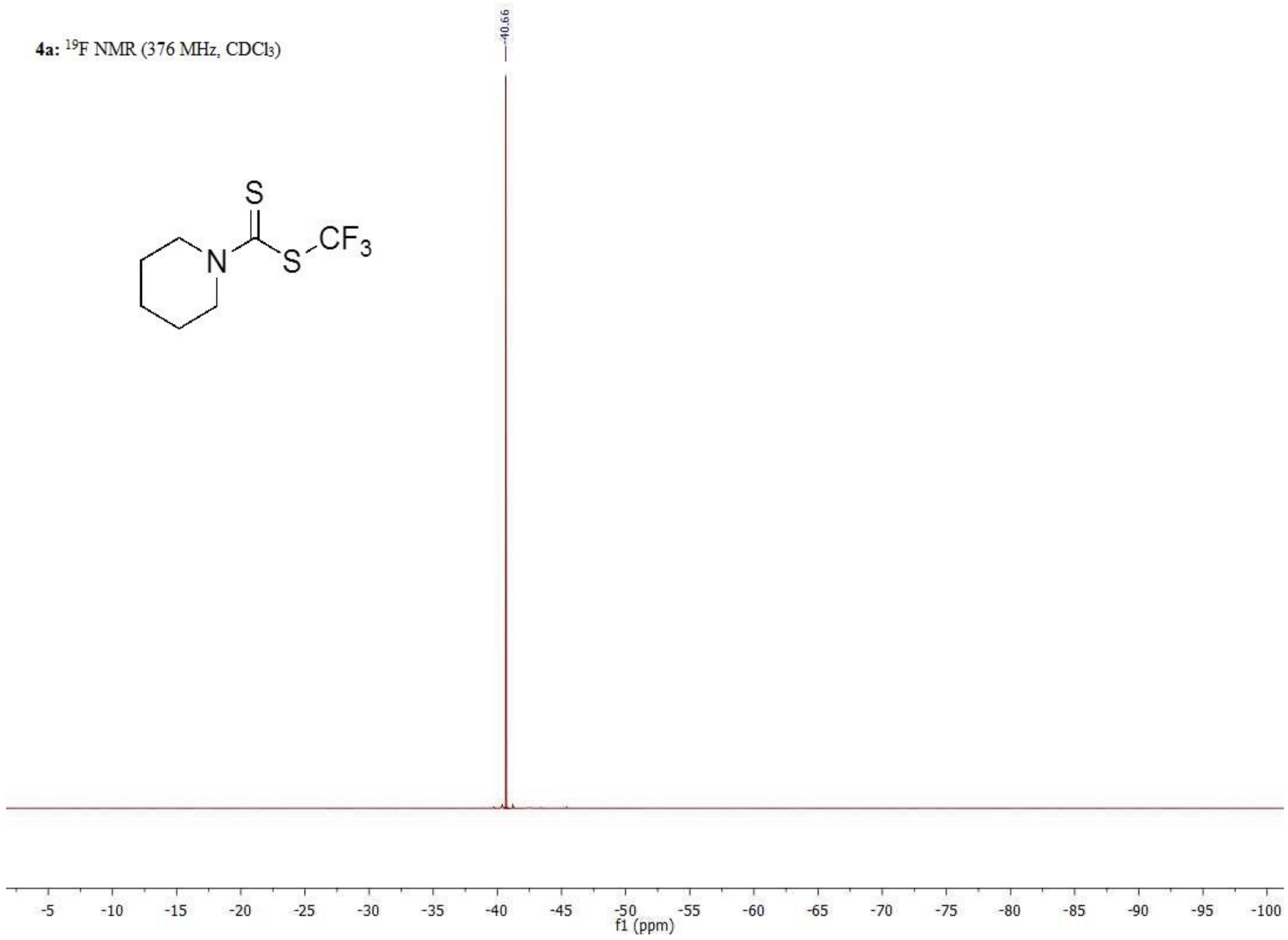

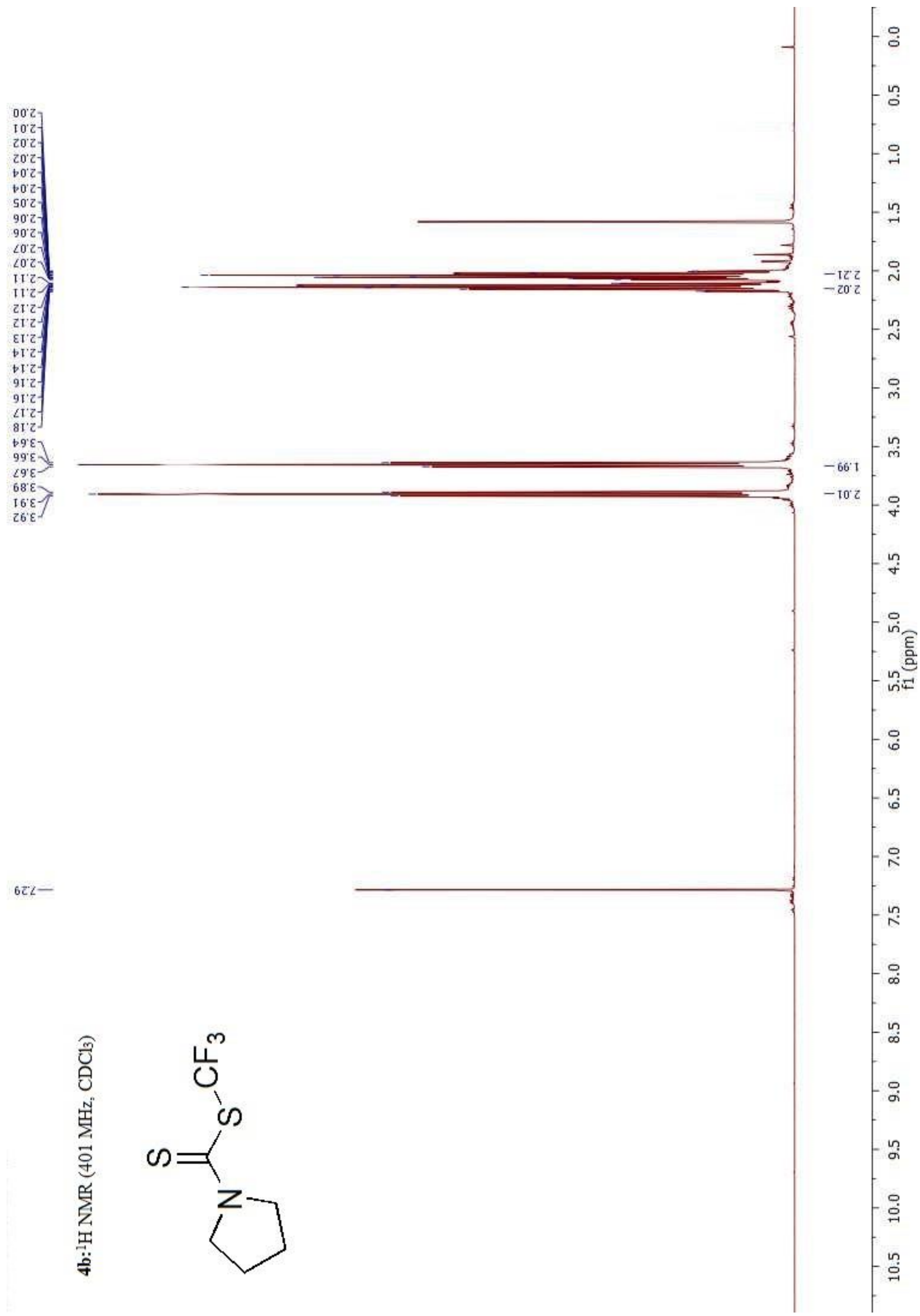
4b: ¹H NMR (401 MHz, CDCl3)
S
N
S
CF3
2.00
2.01
2.02
2.02
2.04
2.04
2.05
2.06
2.06
2.07
2.07
2.11
2.11
2.12
2.12
2.13
2.14
2.14
2.16
2.16
2.17
2.18
3.64
3.66
3.67
3.89
3.91
3.92
7.29
2.21
2.02
1.99
2.01
f1 (ppm)

4b: ^{13}C NMR (126 MHz, $CDCl_3$)

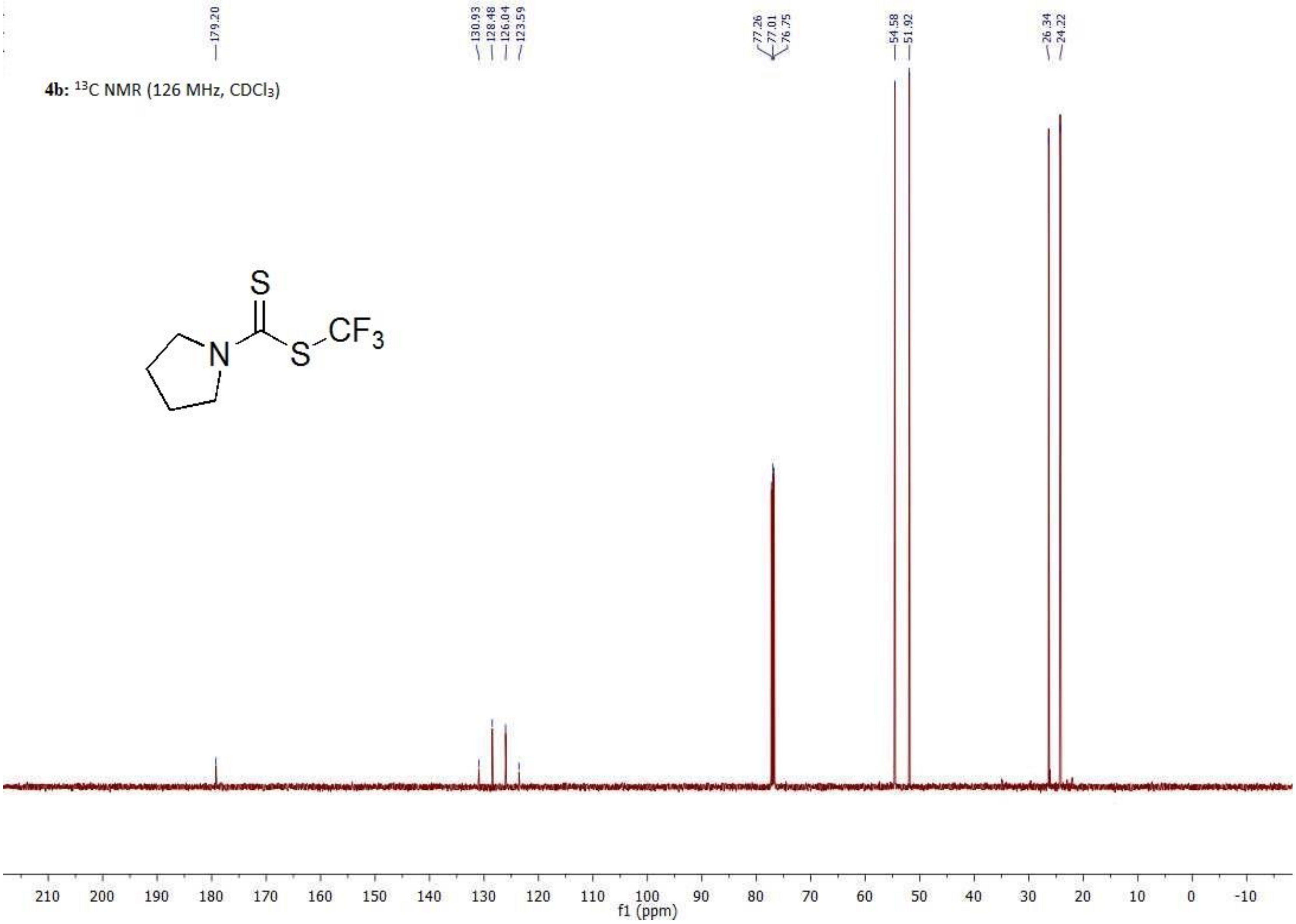

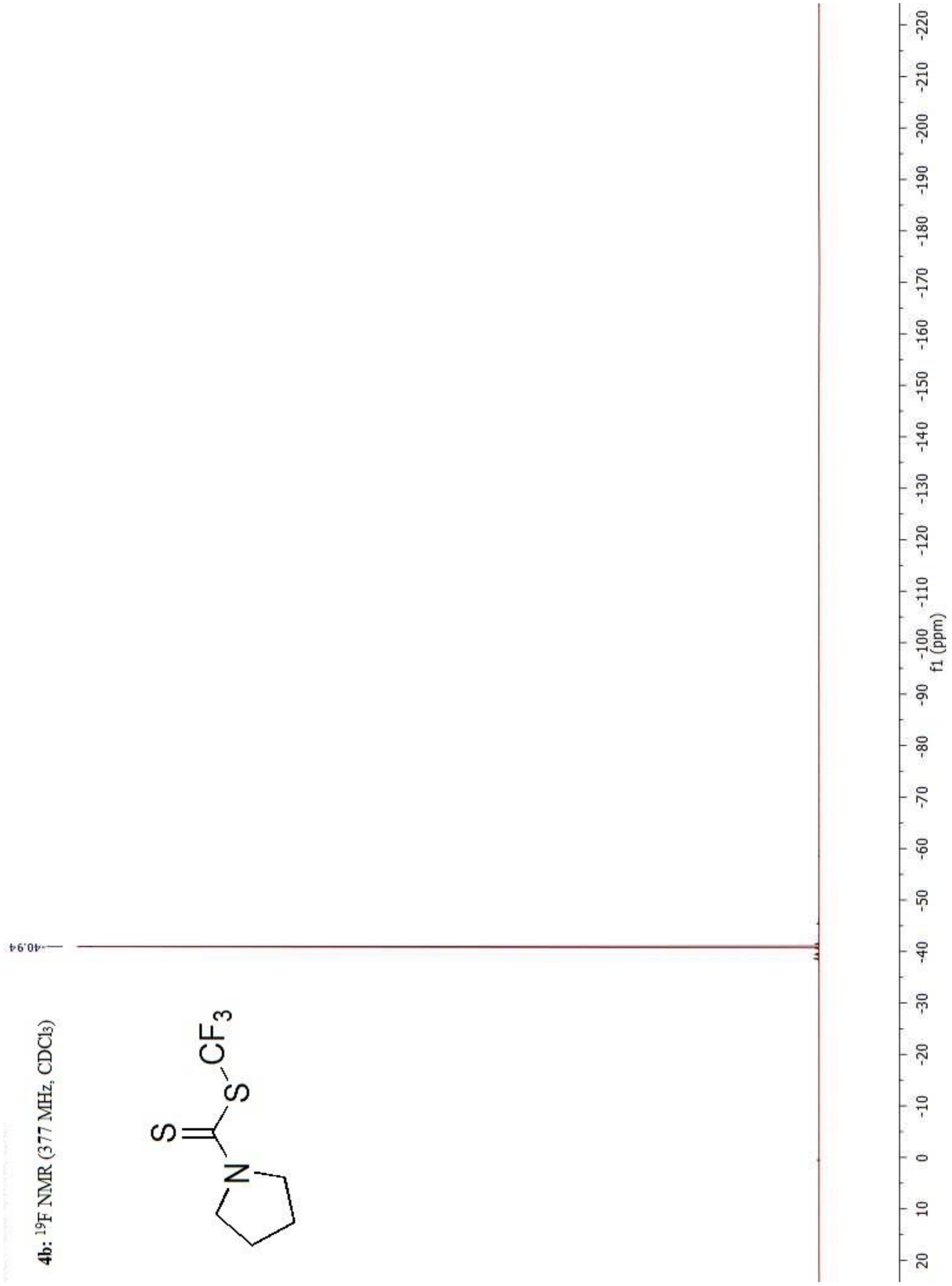

4b: ^{19}F NMR (377 MHz, $CDCl_3$)

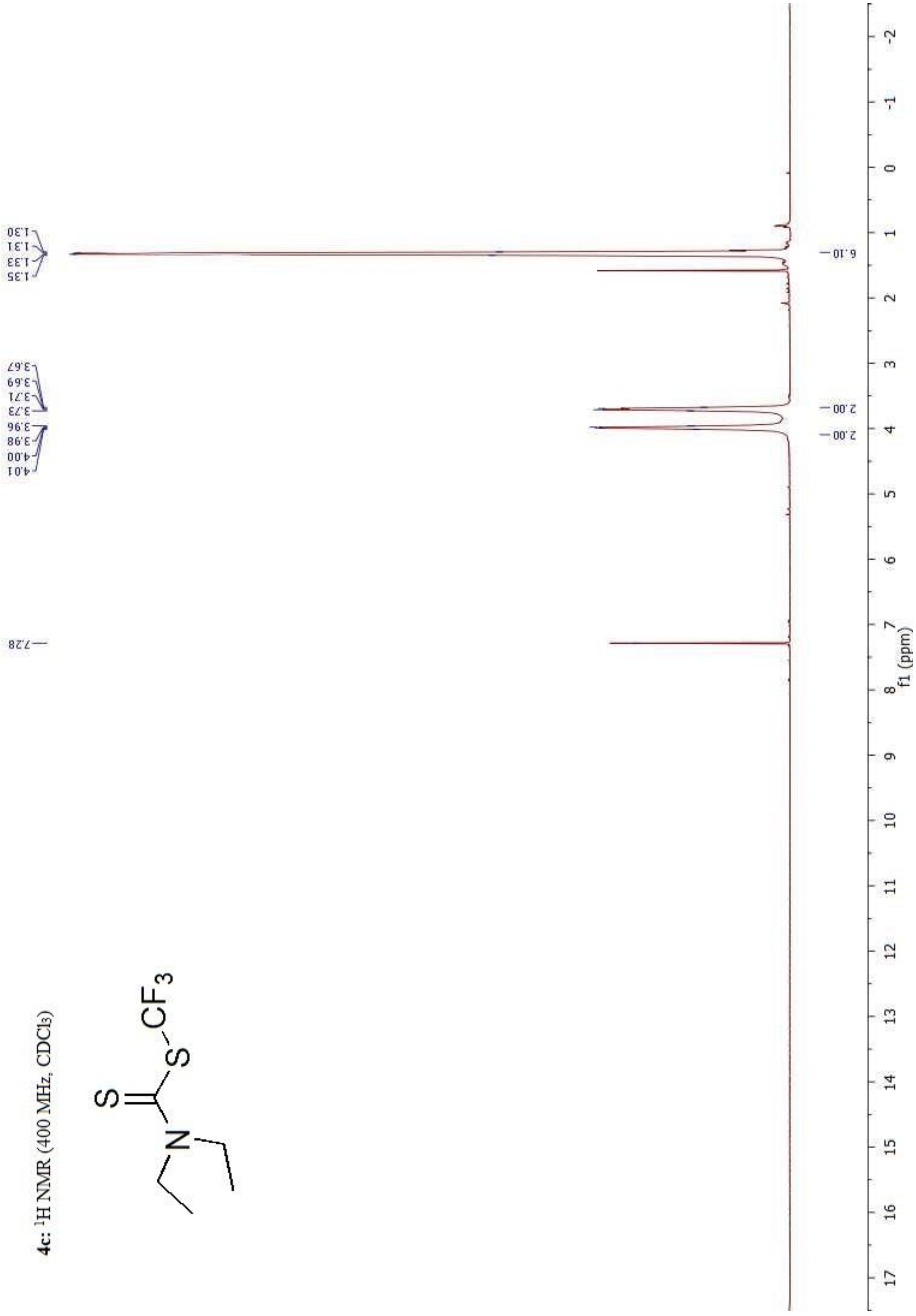

4c: ^{1}H NMR (400 MHz, $CDCl_3$)

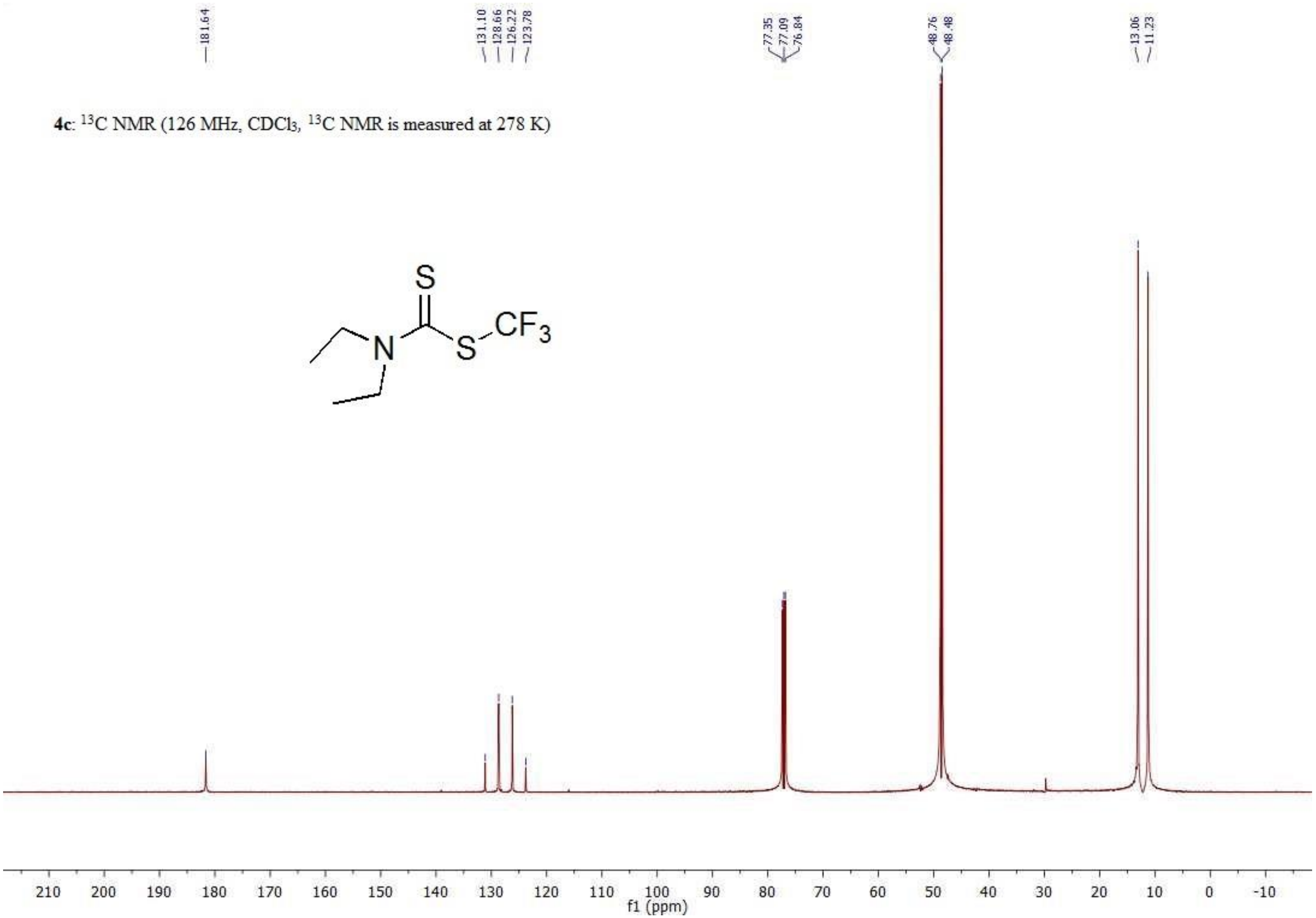

4c: ^{13}C NMR (126 MHz, $CDCl_3$, ^{13}C NMR is measured at 278 K)

4c: ^{19}F NMR (376 MHz, $CDCl_3$)

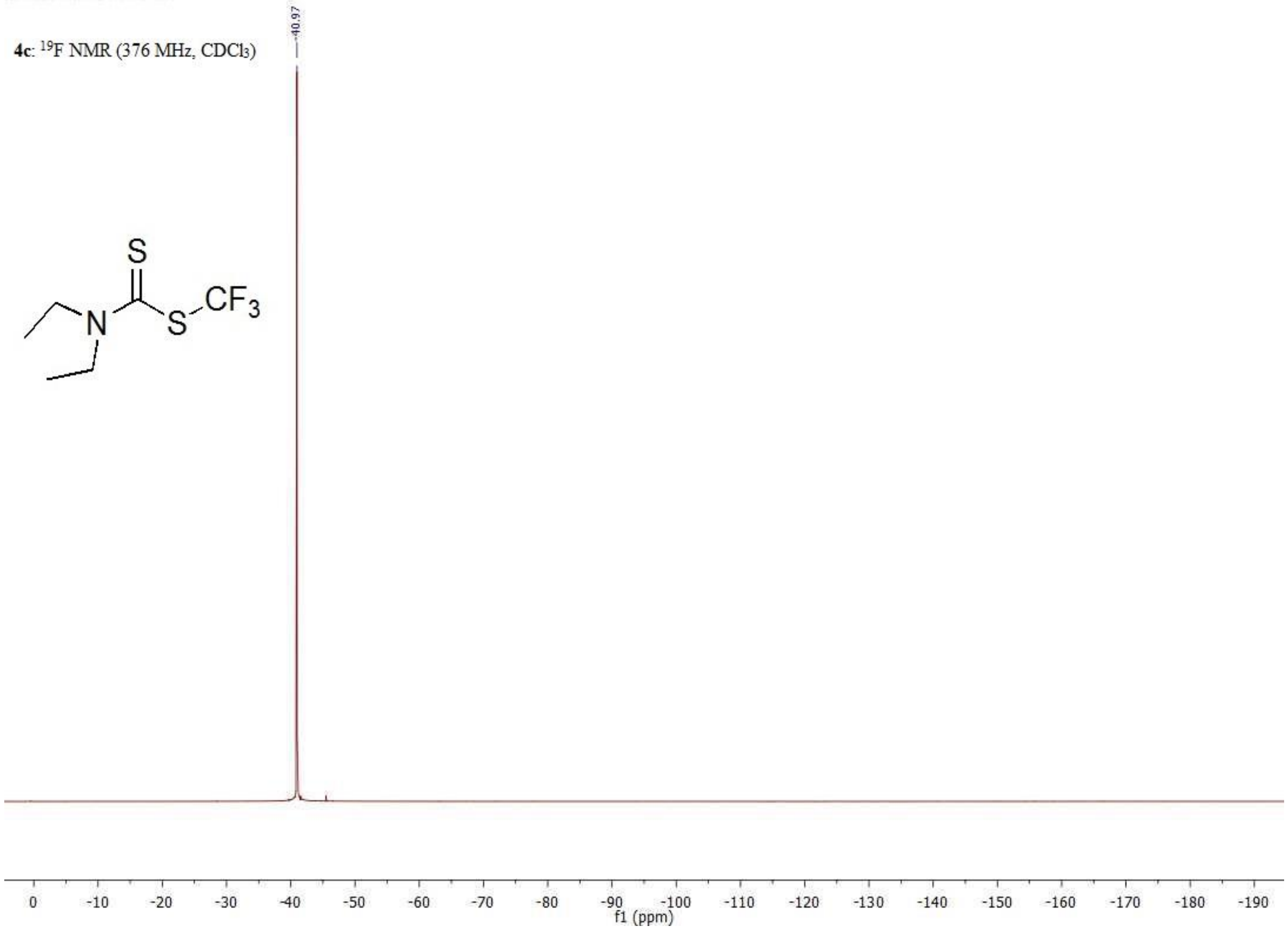

4d: ¹H NMR (400 MHz, $CDCl_3$)

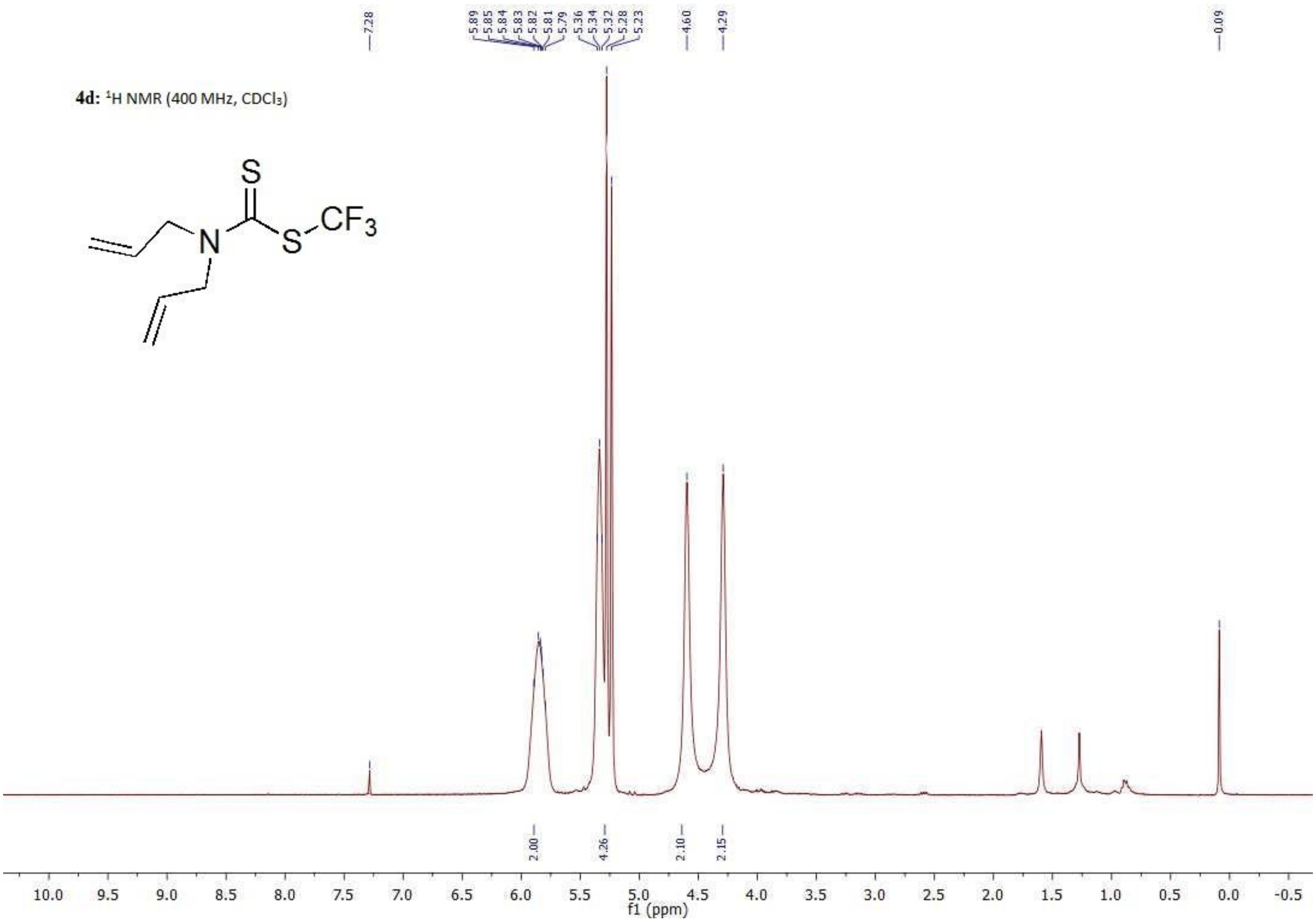

4d: ^{13}C NMR (101 MHz, $CDCl_3$)

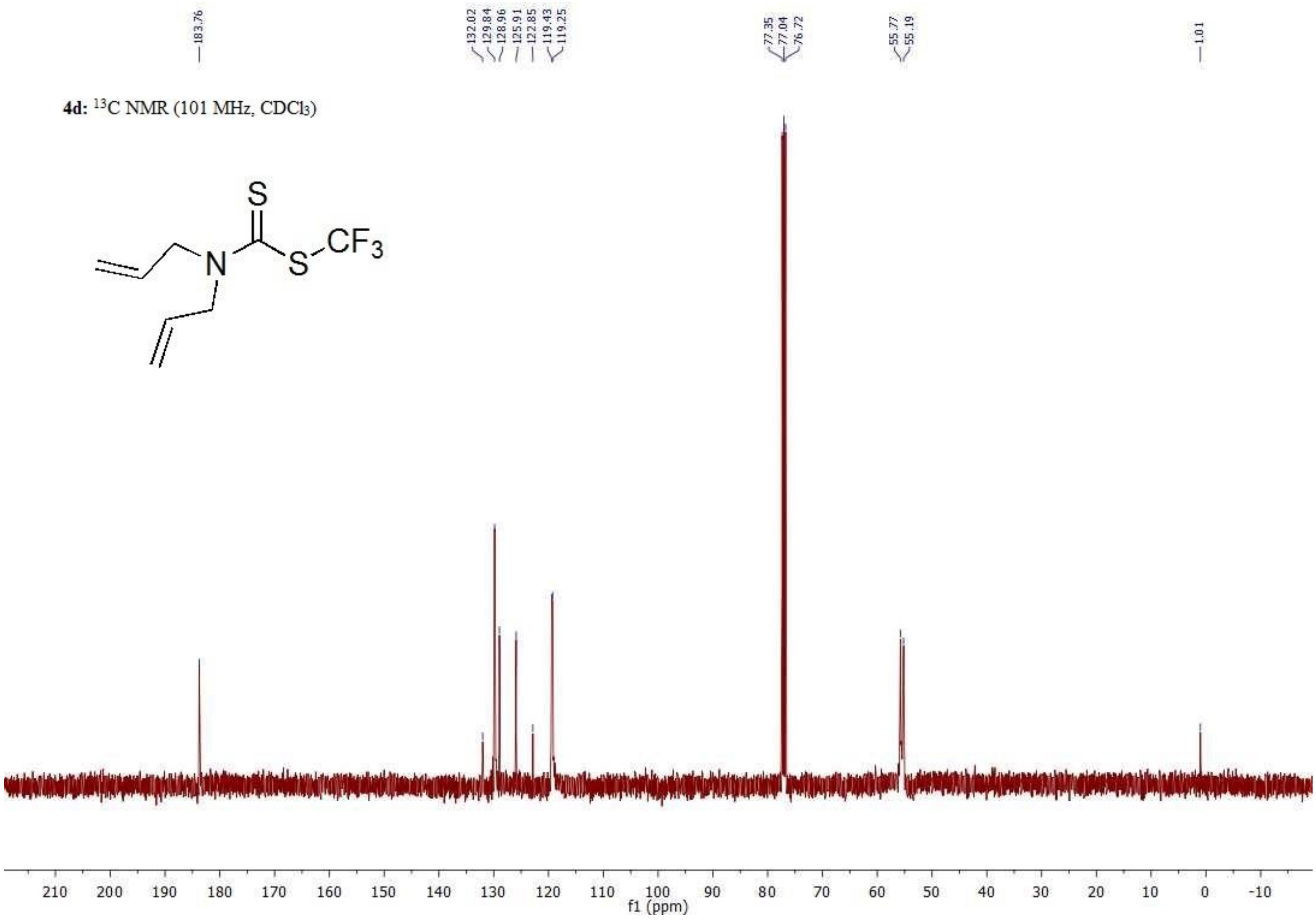

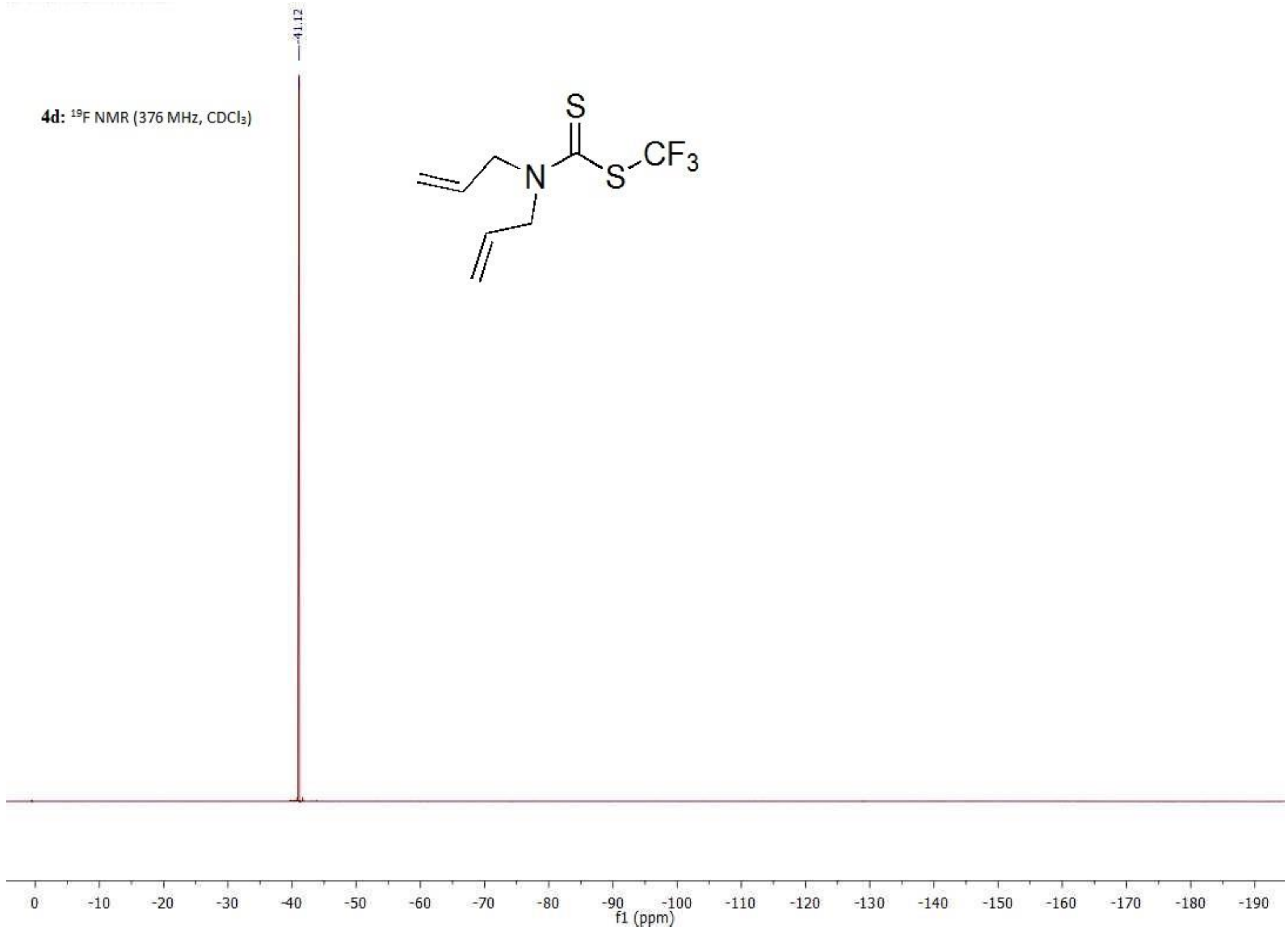

4d: ^{19}F NMR (376 MHz, $CDCl_3$)

4e: ^{1}H NMR (400 MHz, $CDCl_3$)

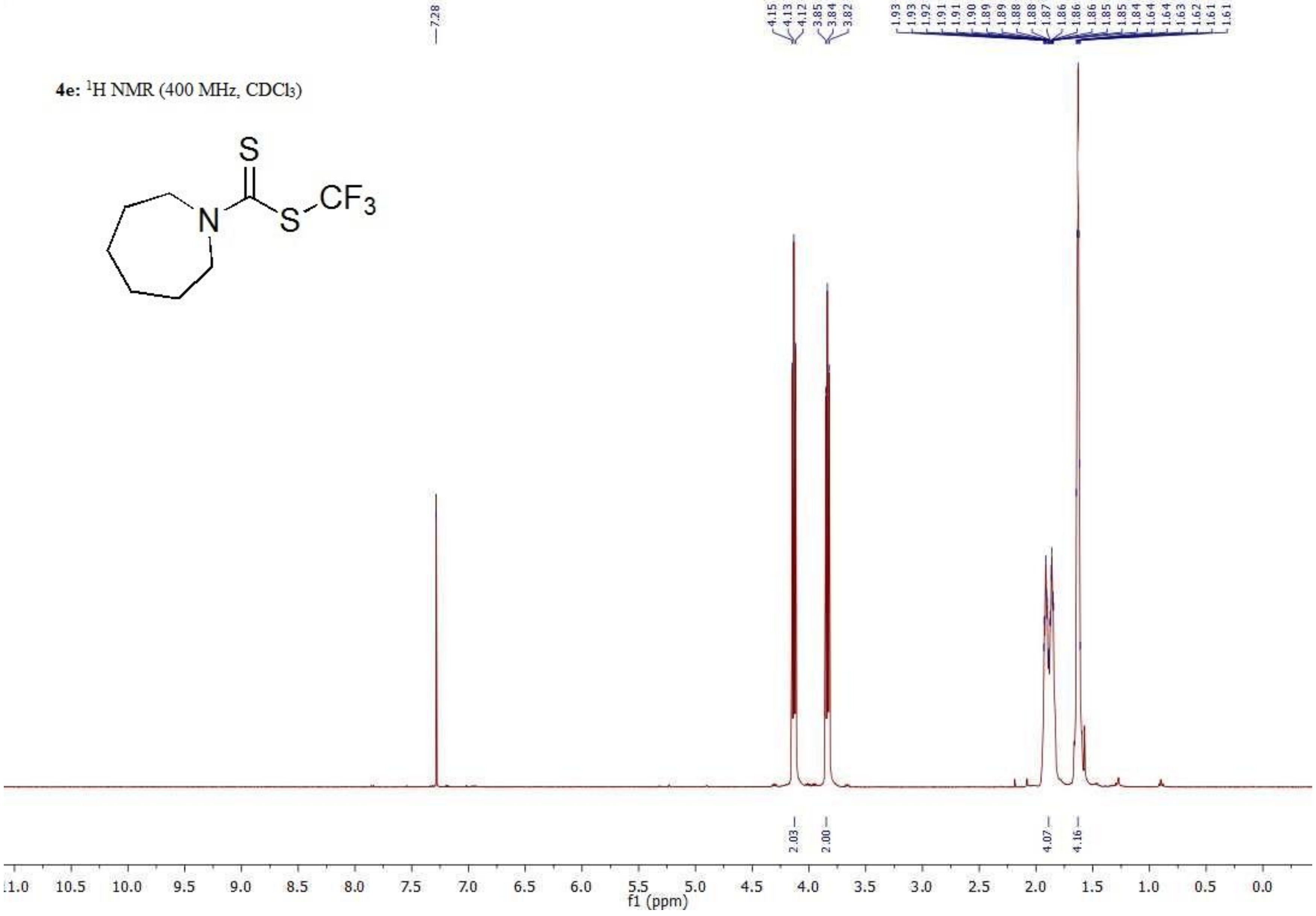

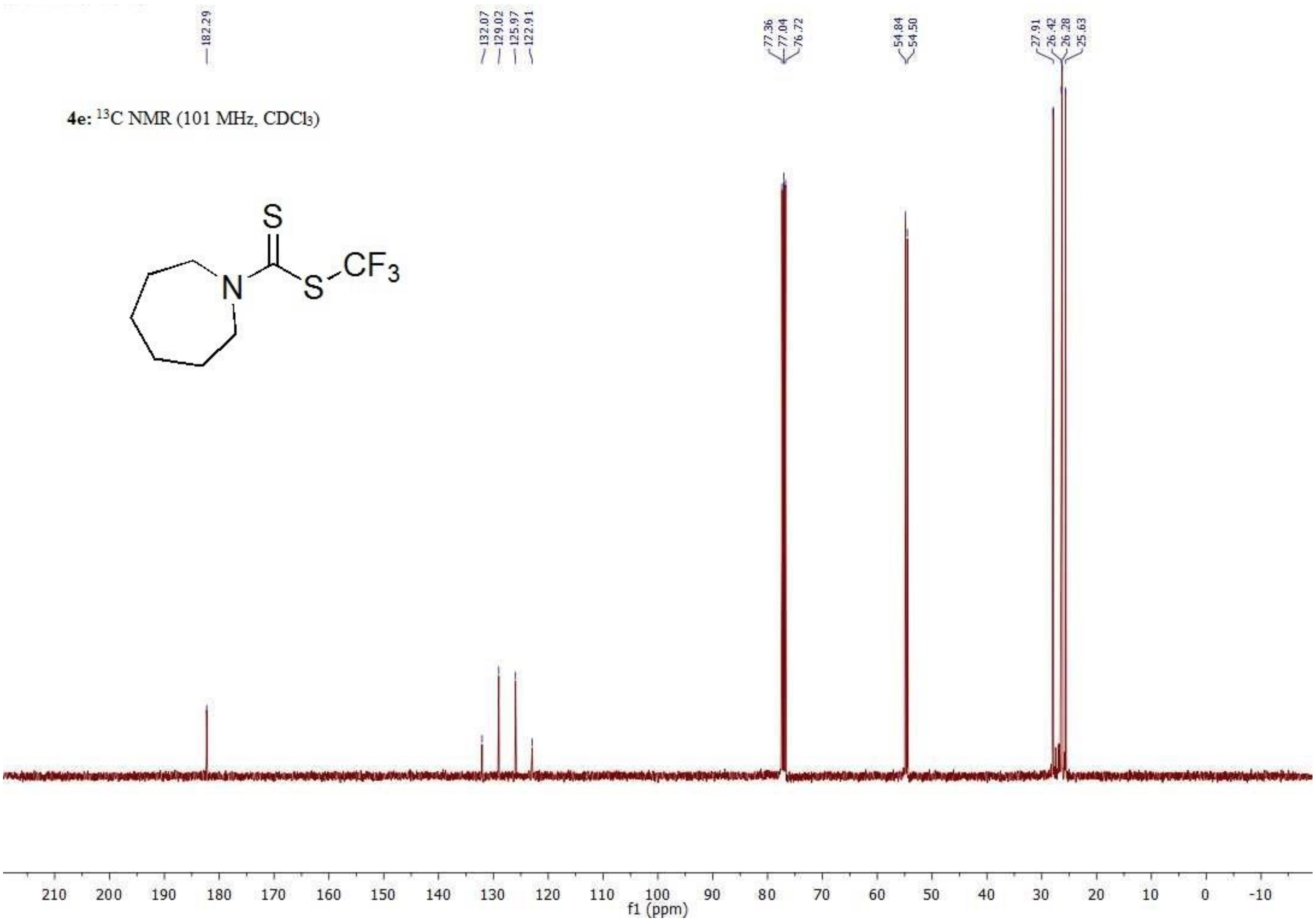

4e: ^{13}C NMR (101 MHz, $CDCl_3$)

4e: ^{19}F NMR (376 MHz, $CDCl_3$)

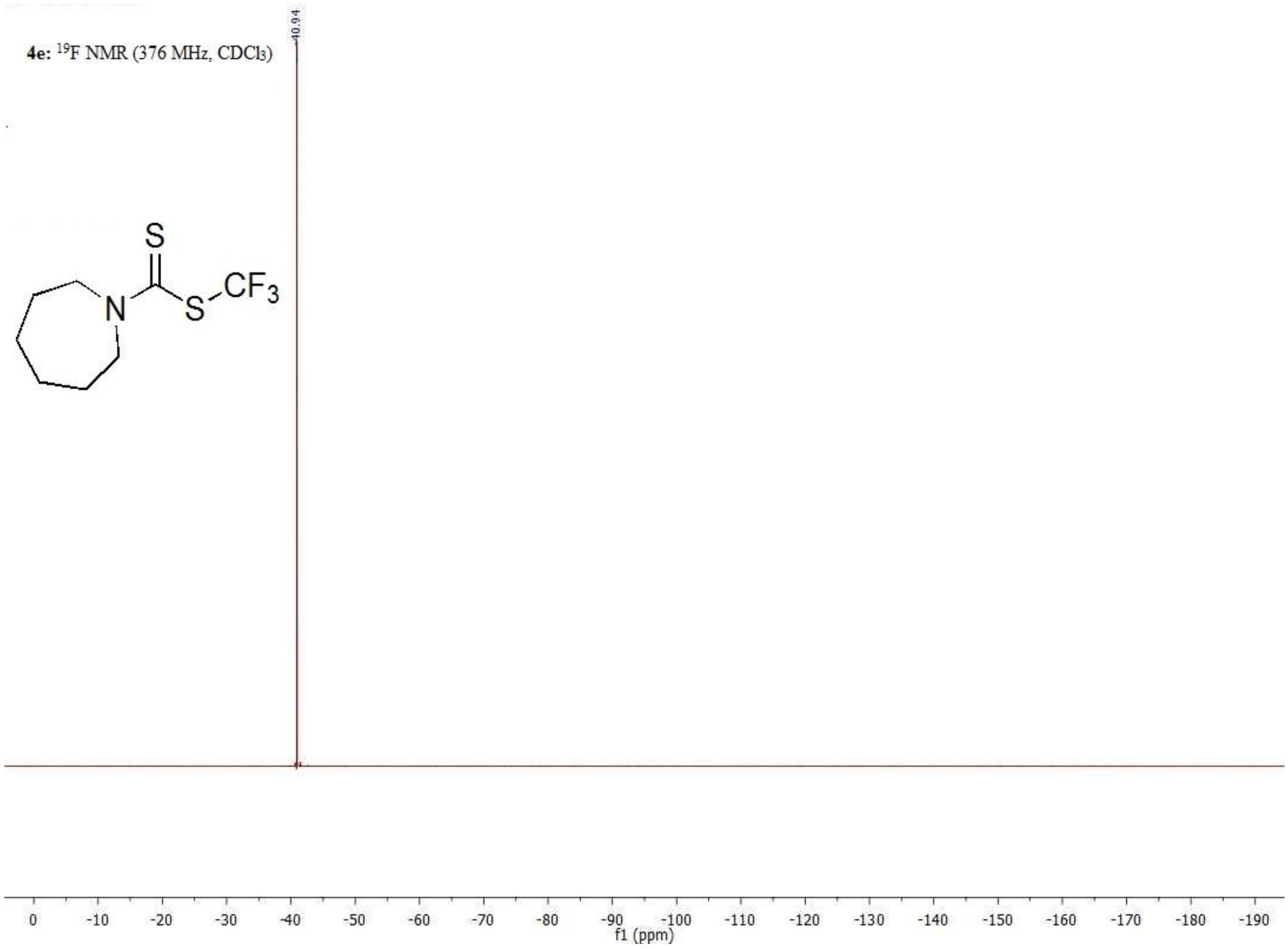

4f: ^{1}H NMR (400 MHz, $CDCl_3$)

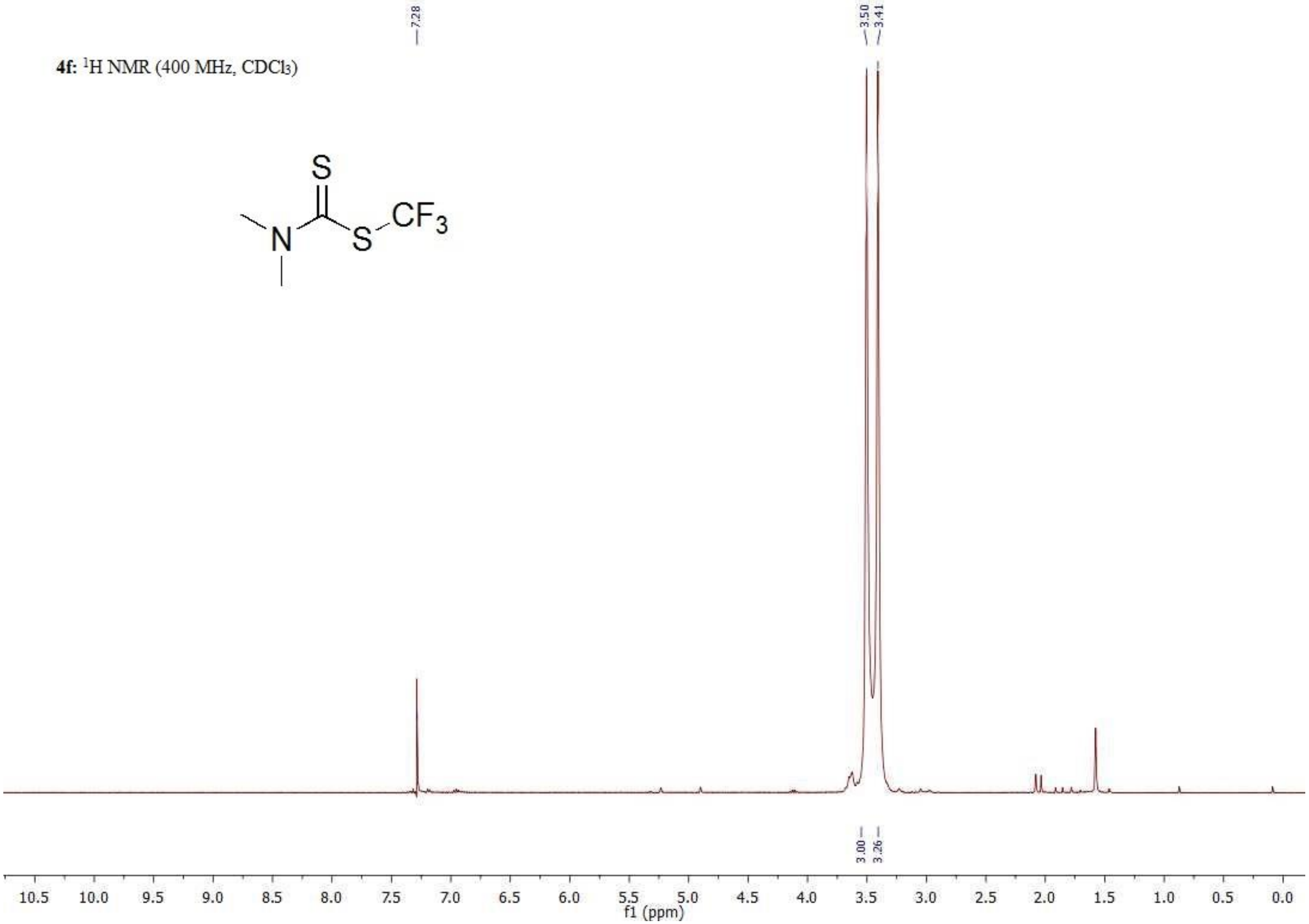

4f: ^{13}C NMR (101 MHz, $CDCl_3$)

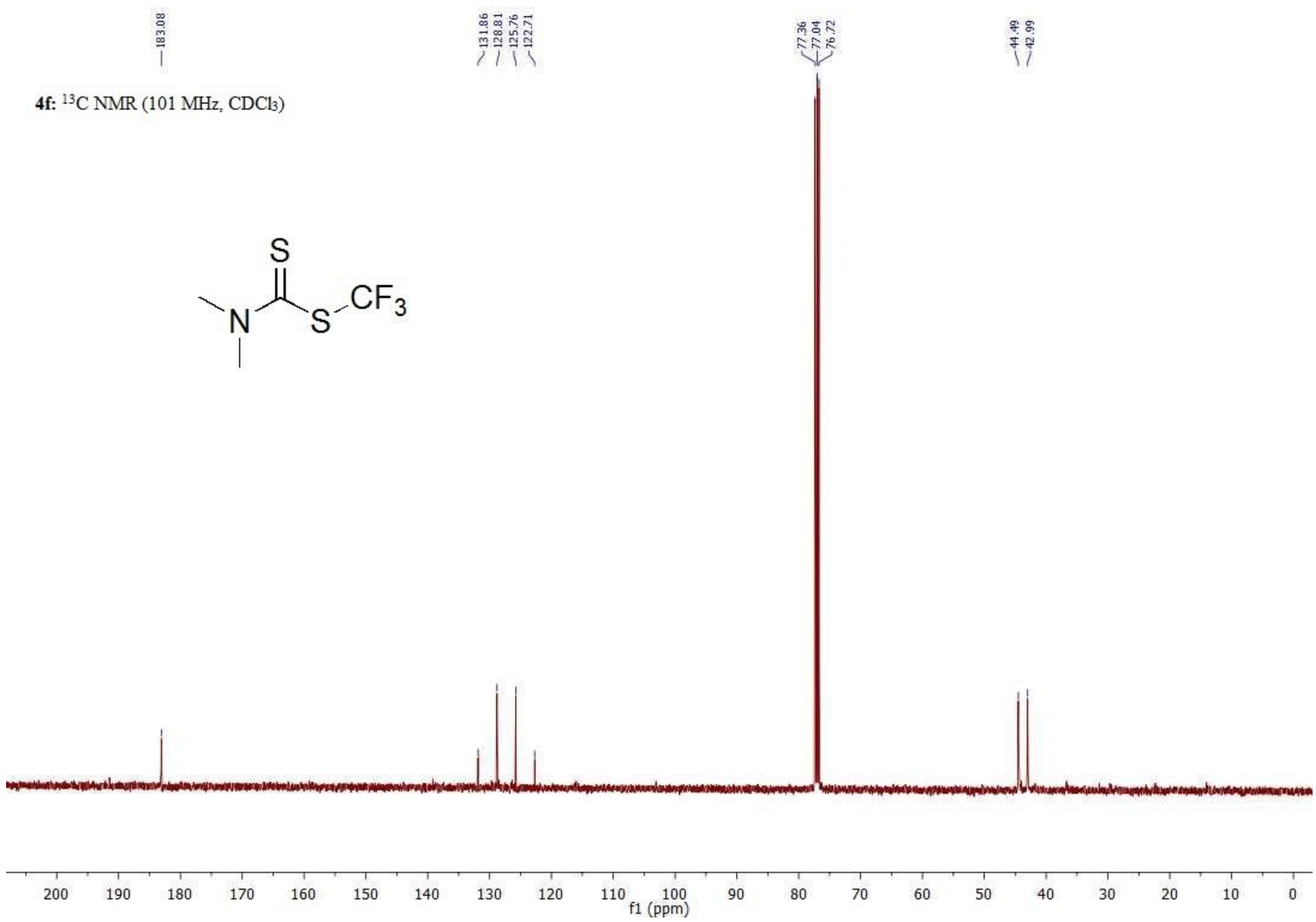

4f: ^{19}F NMR (376 MHz, $CDCl_3$)

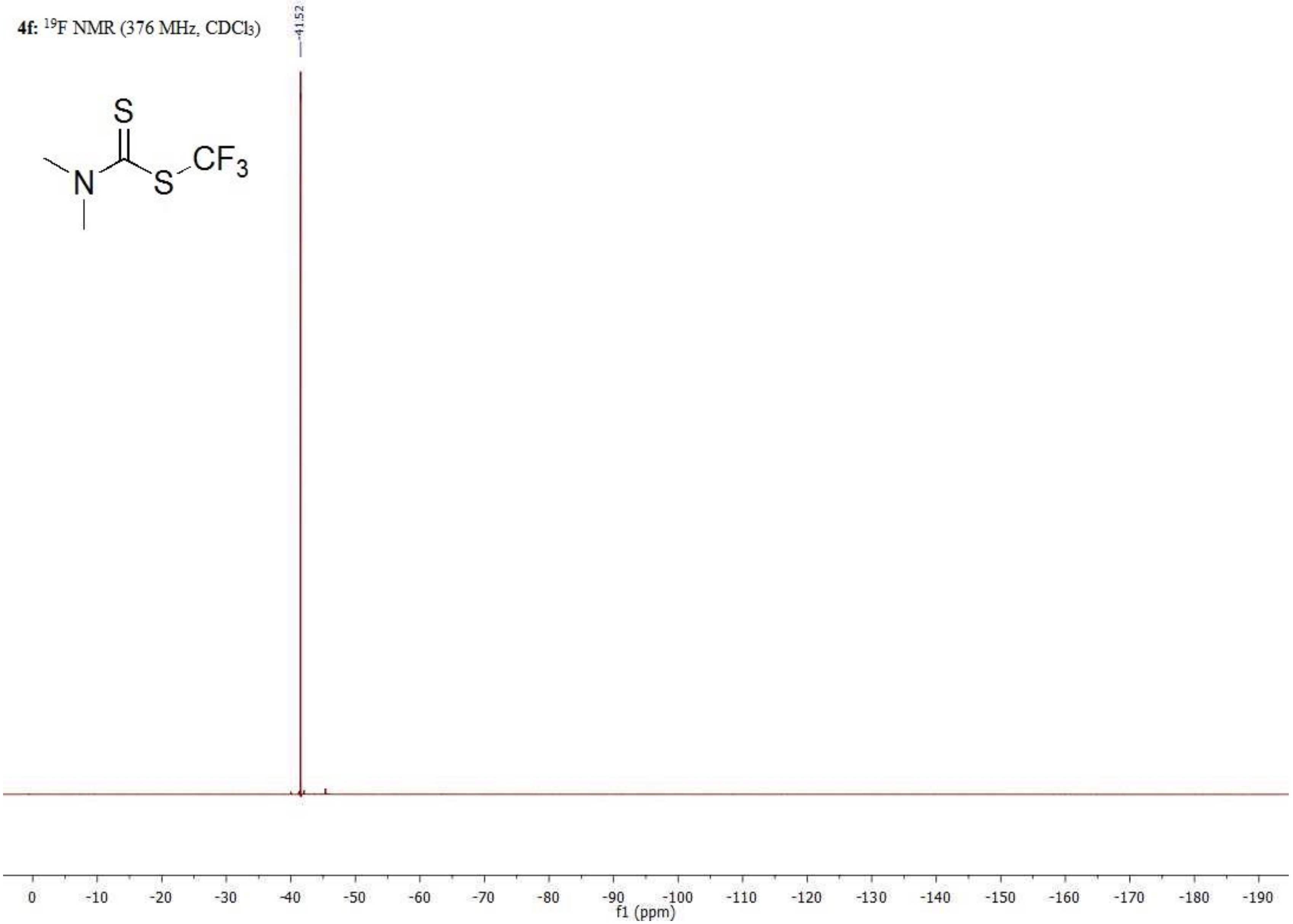

4g: ^{1}H NMR (400 MHz, $CDCl_3$)

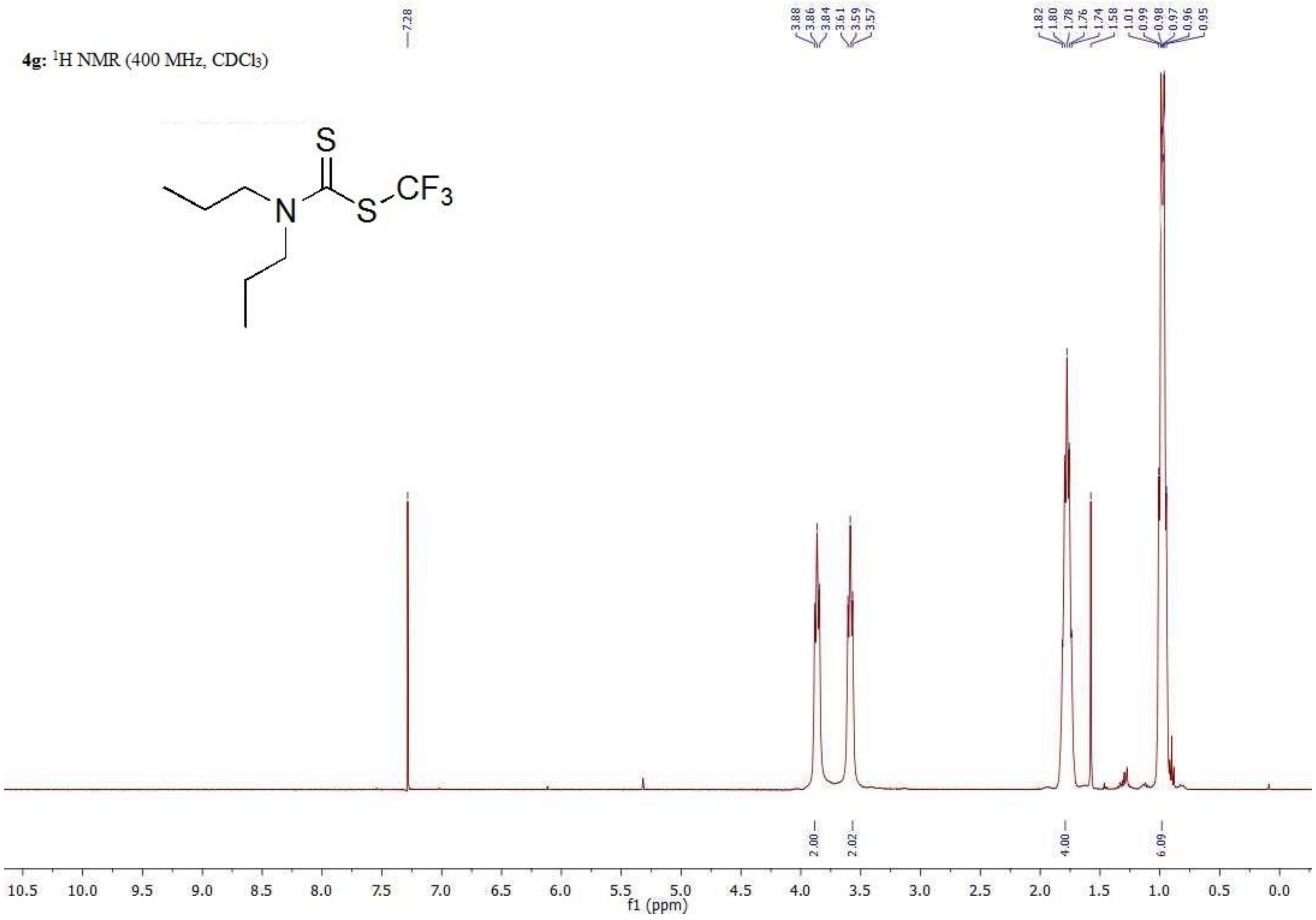

4g: ^{13}C NMR (101 MHz, $CDCl_3$)

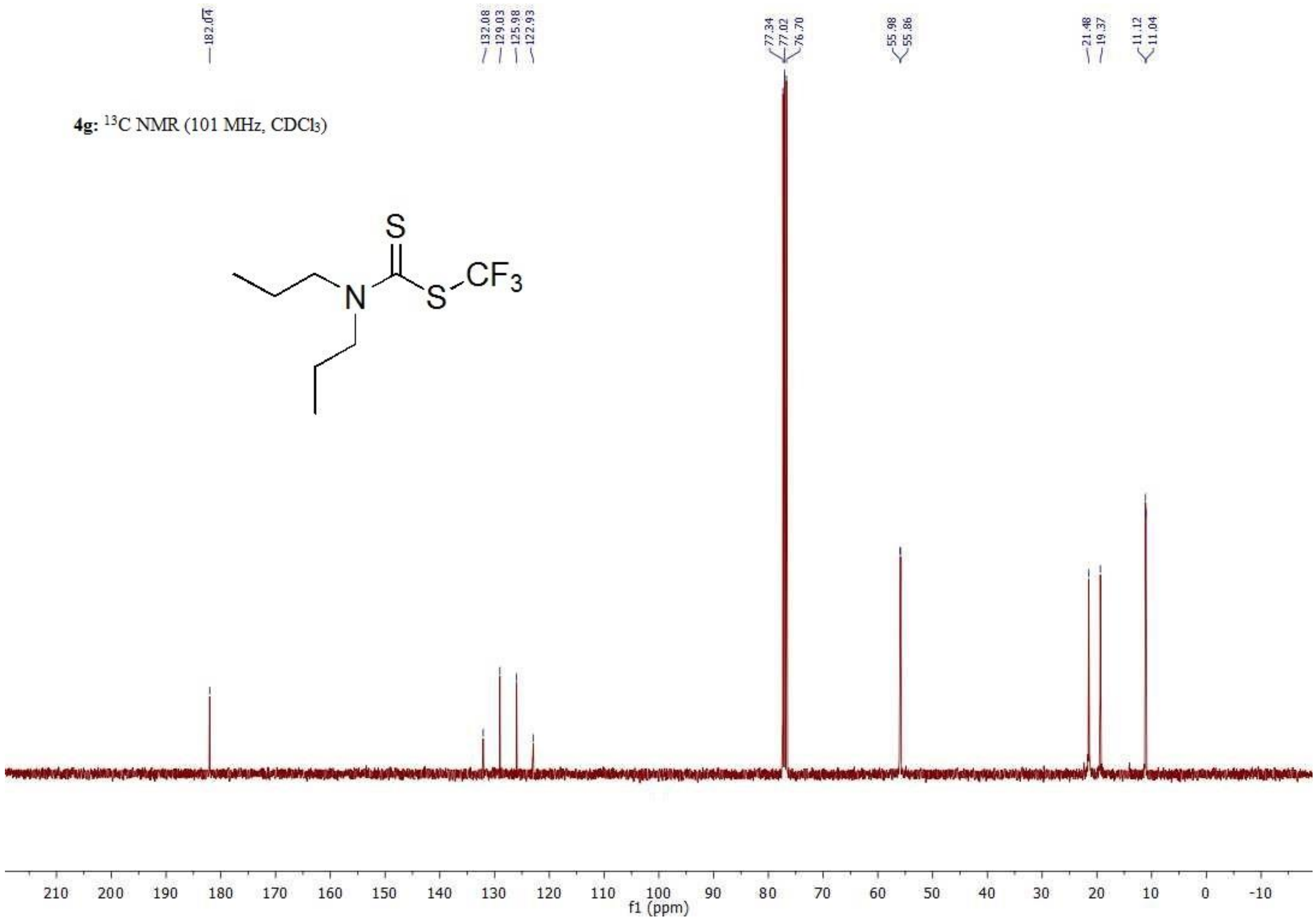

4g: ^{19}F NMR (376 MHz, $CDCl_3$)

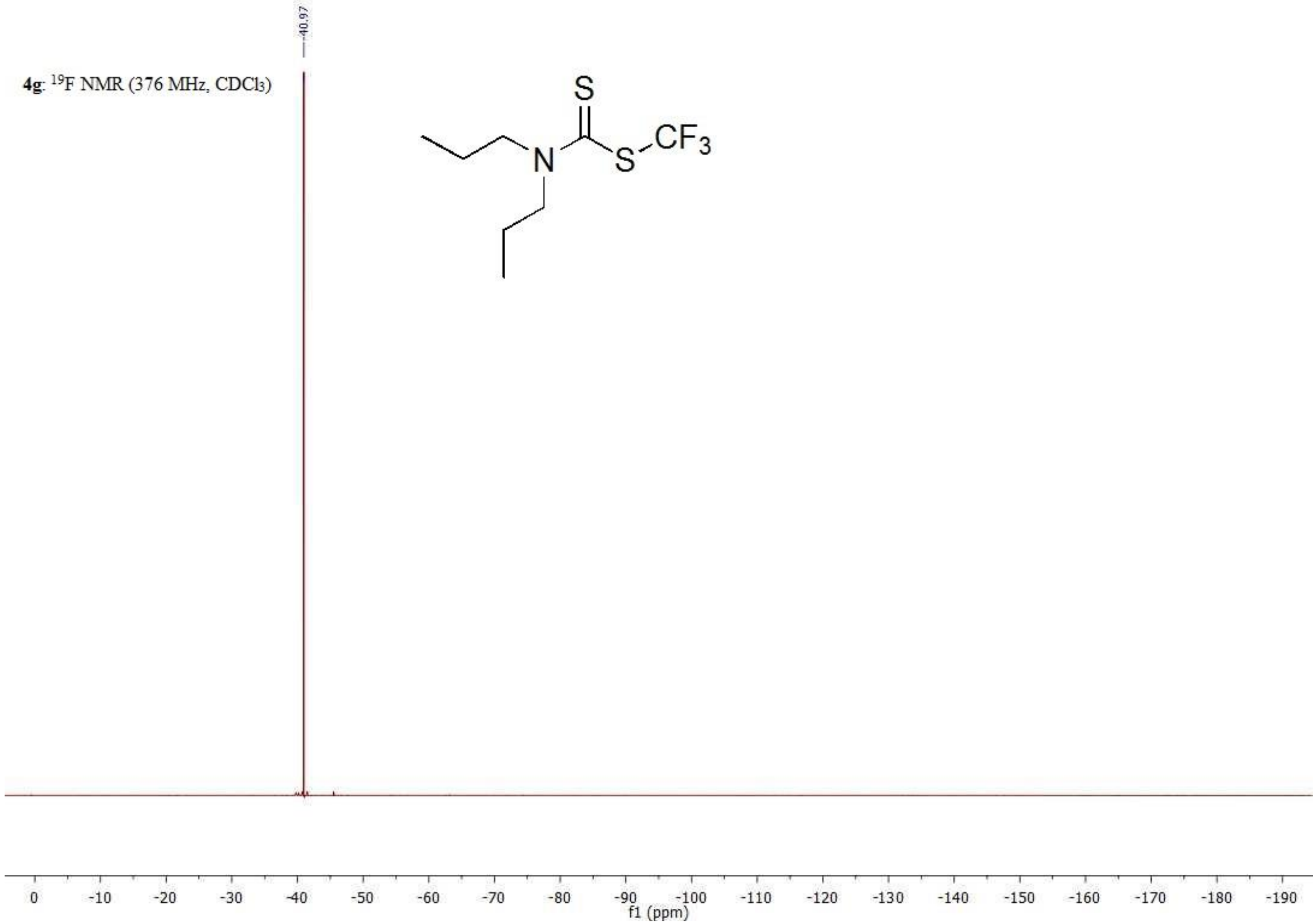

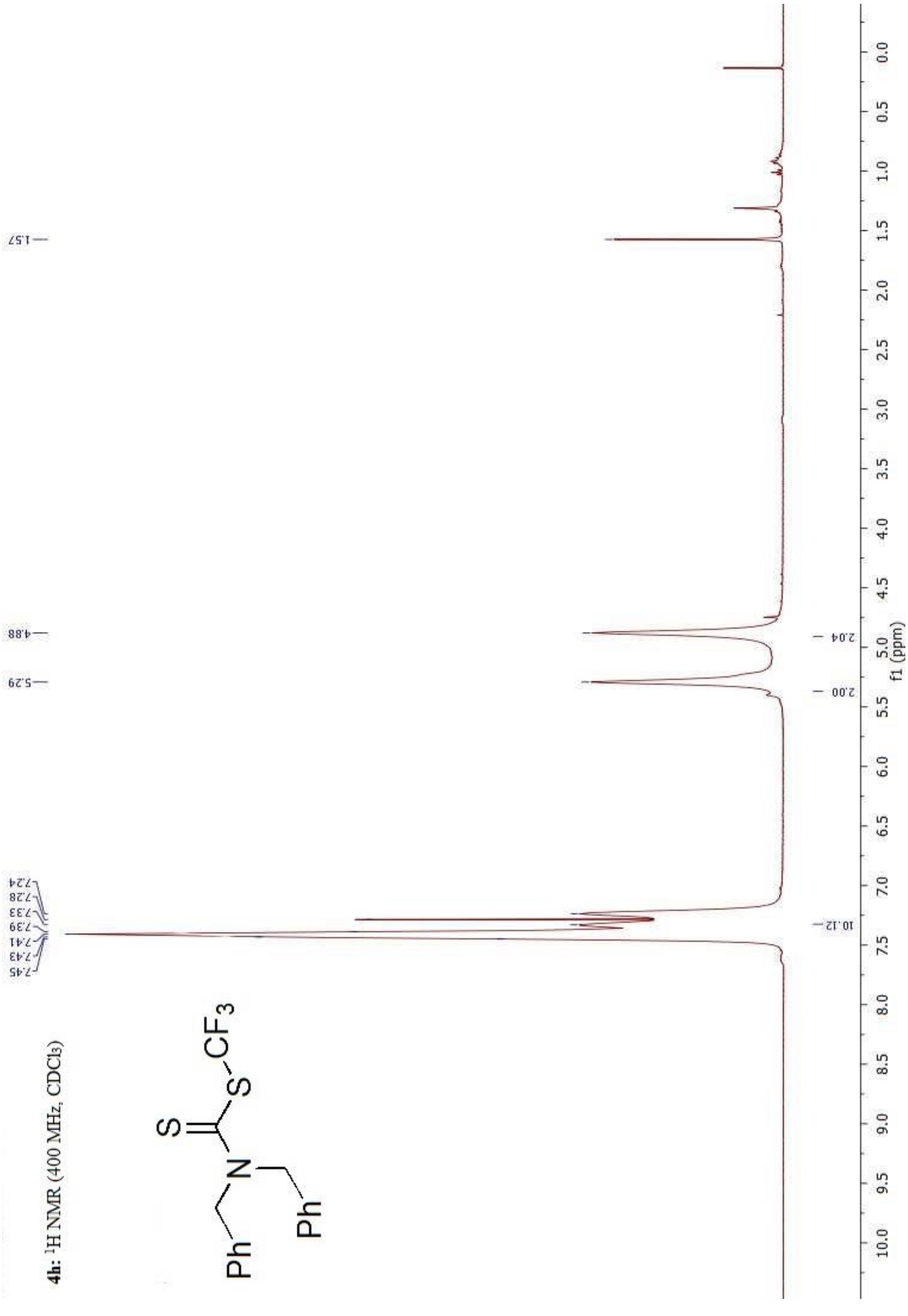
4h: 1H NMR (400 MHz, CDCl3)
7.45
7.43
7.41
7.39
7.33
7.28
7.24
5.29
4.88
1.57
10.12
2.00
2.04
f1 (ppm)

4h: ^{13}C NMR (101 MHz, $CDCl_3$)

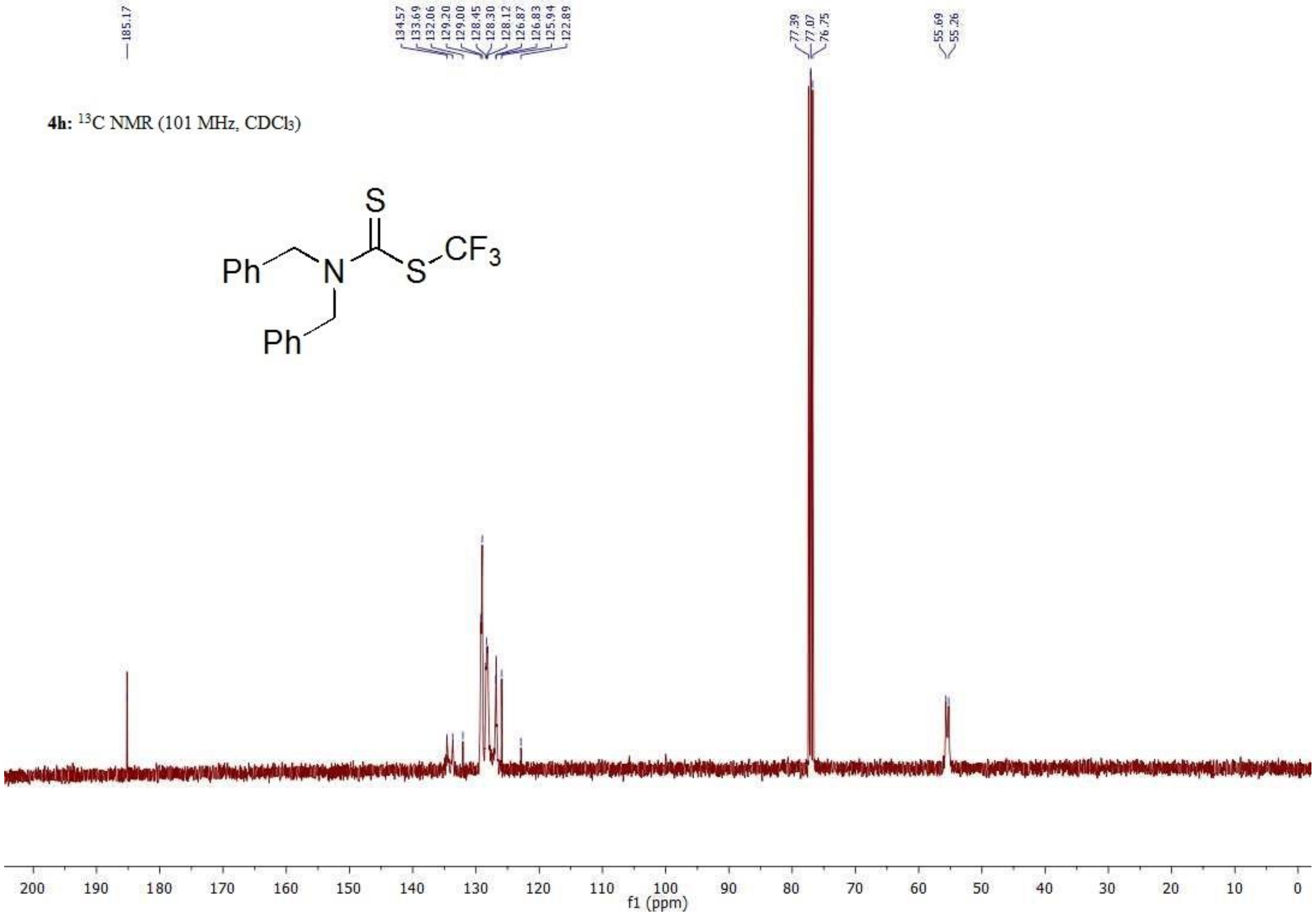

4h: ^{19}F NMR (376 MHz, $CDCl_3$)

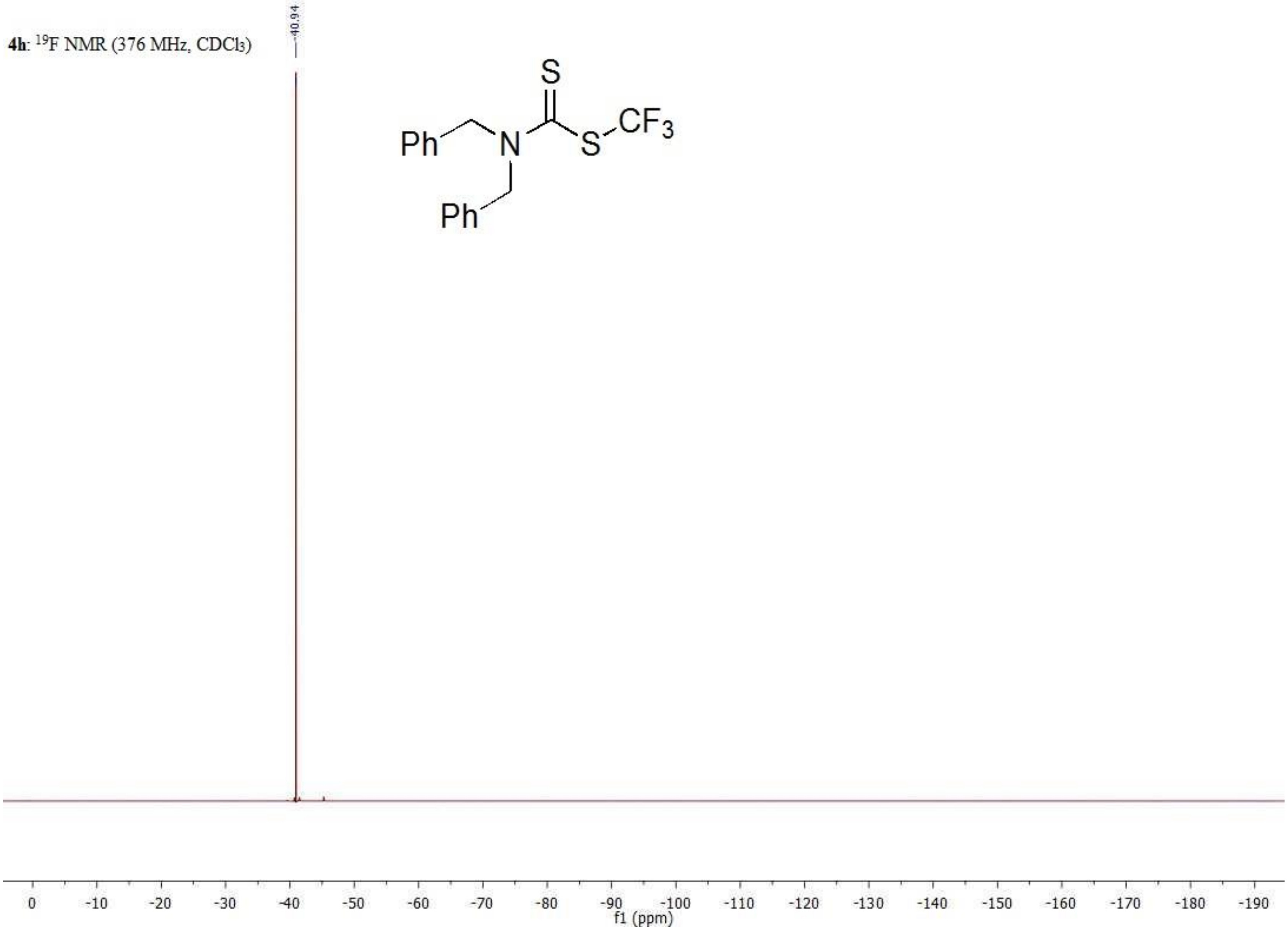

4i: ^{1}H NMR (400 MHz, $CDCl_3$)

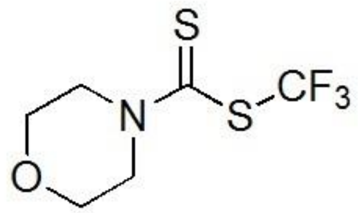

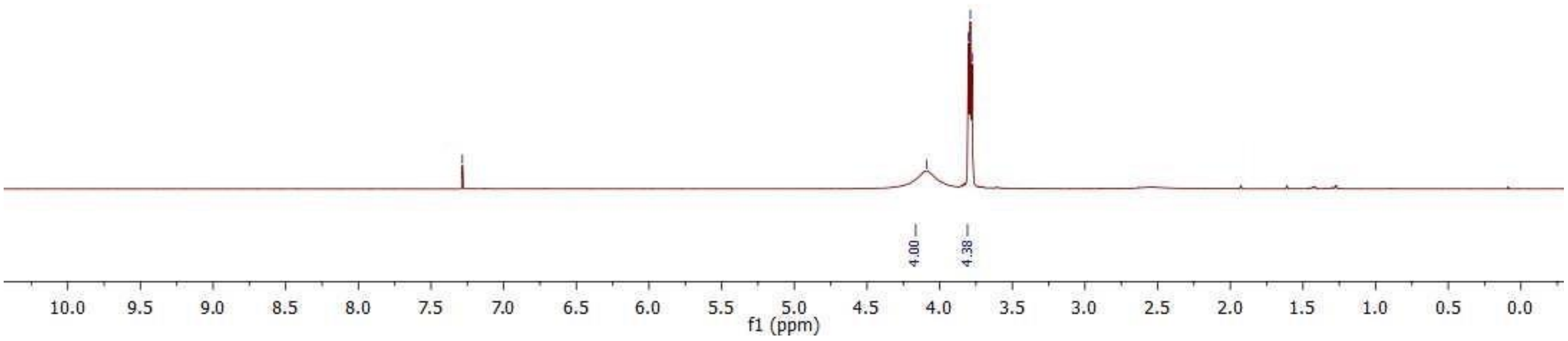

4i: ^{13}C NMR (101 MHz, $CDCl_3$)

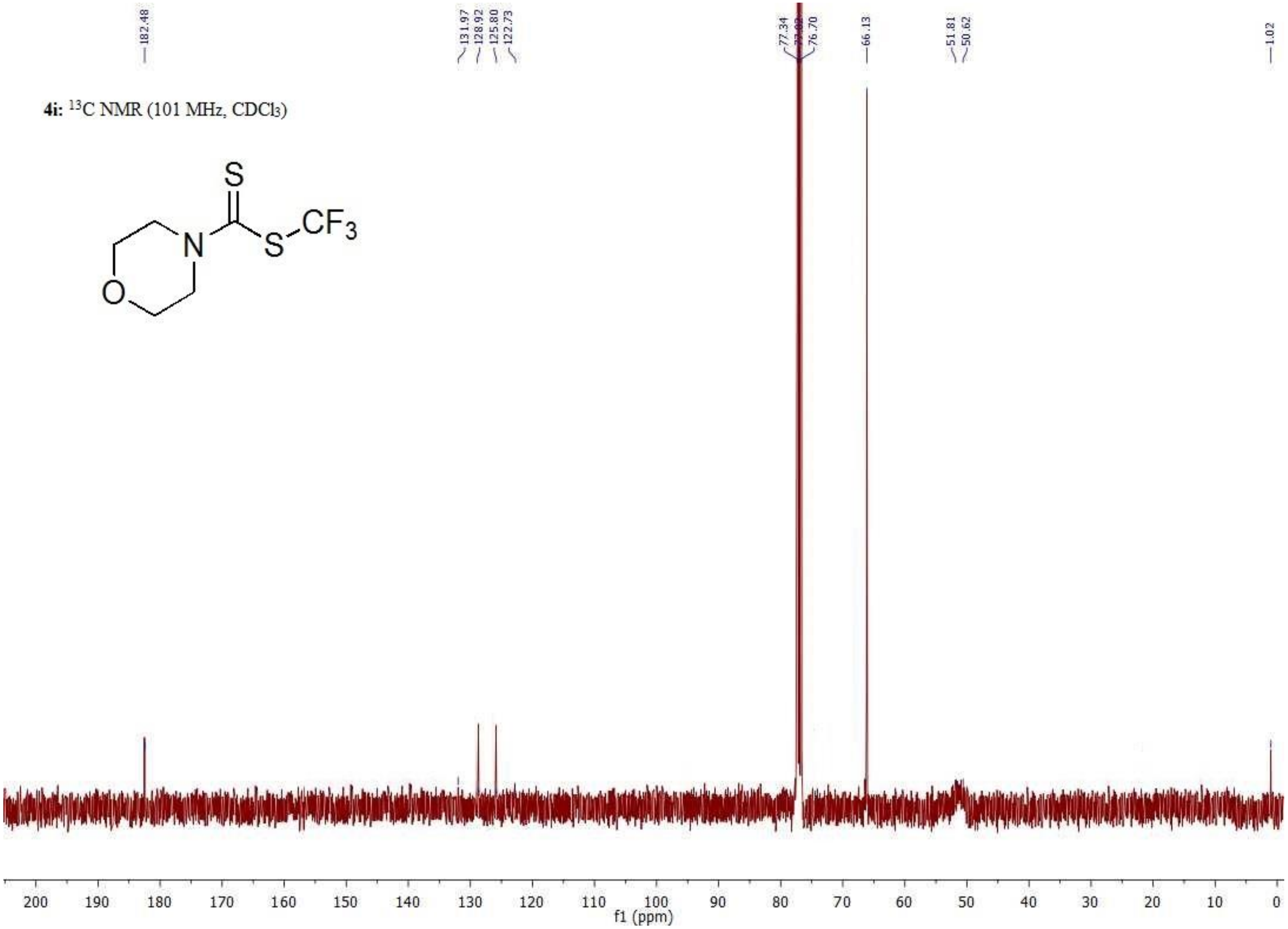

4i: ^{19}F NMR (376 MHz, $CDCl_3$)

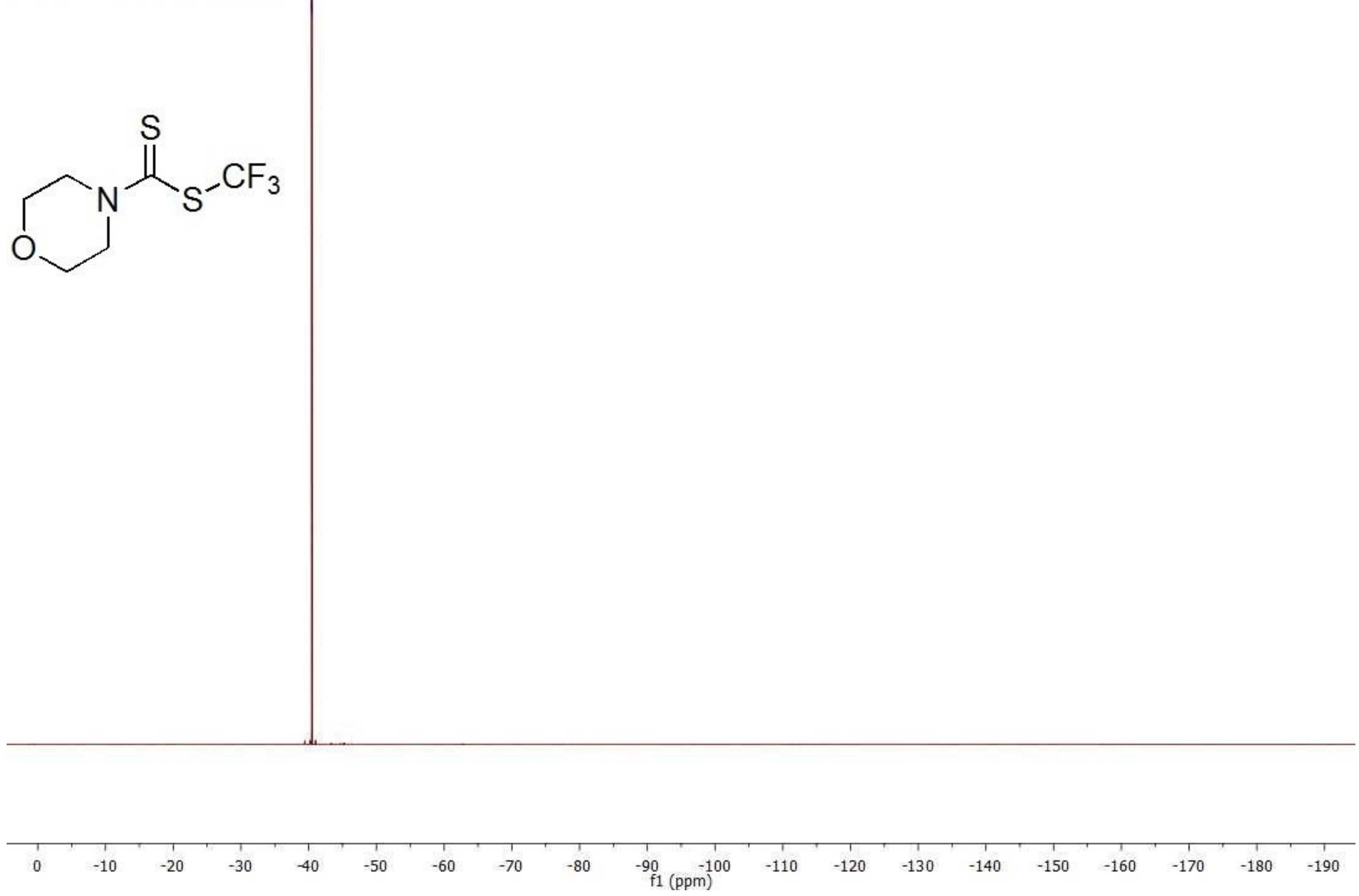

benzylisothiocyanate: ^{1}H NMR (400 MHz, $CDCl_3$)

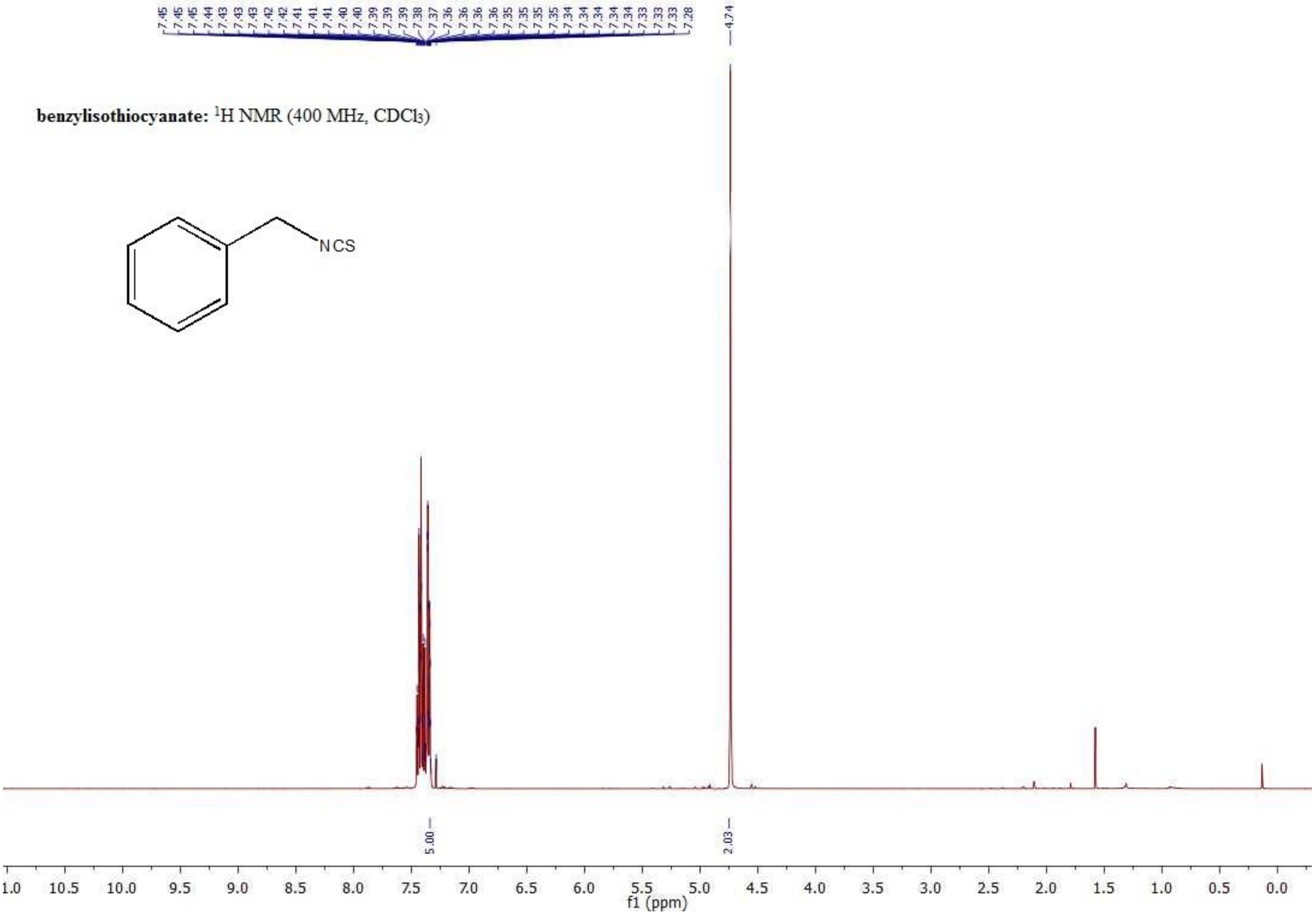

benzylisothiocyanate: ^{13}C NMR (101 MHz, $CDCl_3$)

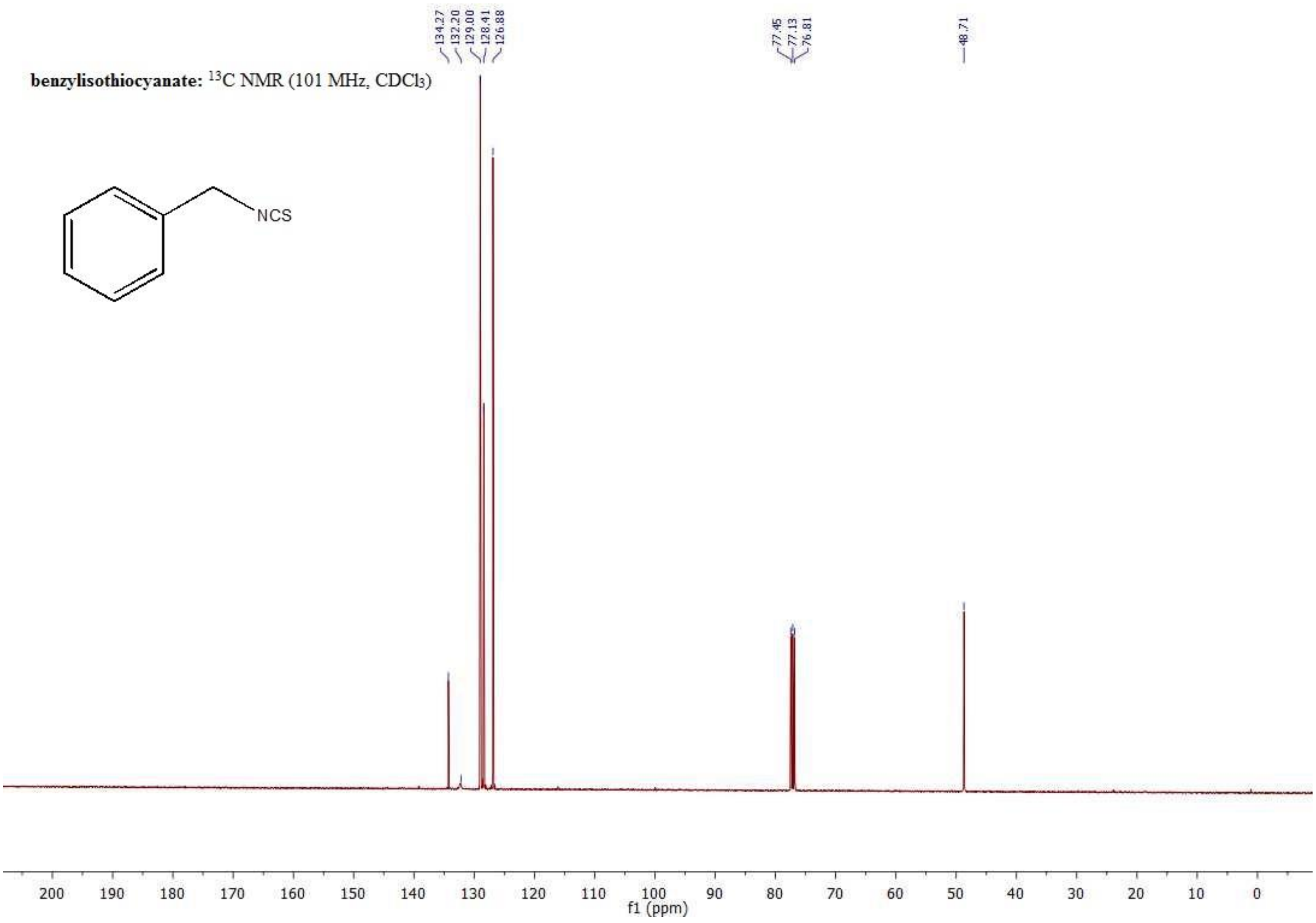

4. DNMR and DFT calculations

Variable-temperature NMR experiments were performed on a Bruker spectrometer operating at 499.9 MHz for ^{1}H and at 125.7 MHz for ^{13}C.

The studied structures were subjected to geometry optimization at DFT level, using B3LYP functional [1-2] and standard 6-31+G(d,p) basis set. The Gaussian16 program package was used throughout this study [3]. The QST3 optimization method [4-5] was applied in the search for the transition state structures of the rotamer interconversion, that is the structures of the reactant, product, and estimated transition state were used as input for the TS search. The vibrational frequencies and free energies were calculated for all of the optimized structures, and the stationary-point character (a minimum or a first-order saddle point) was thus confirmed.

5. References

1. Becke, A. D. *J. Chem. Phys.* **1993,** *98*, 5648-5652. doi: 10.1063/1.464913
2. Lee, C. T.; Yang, W. T.; Parr, R. G. *Phys. Rev. B* **1988,** *37*, 785-789. doi:10.1103/PhysRevB.37.785
3. Frisch, M. J.; Trucks, G. W.; Schlegel, H. B.; Scuseria, G. E.; Robb, M. A.; Cheeseman, J. R.; Scalmani, G.; Barone, V.; Petersson, G. A.; Nakatsuji, H.; Li, X.; Caricato, X.; Marenich, A. V.; Bloino, J.; Janesko, B. G.; Gomperts, R.; Mennucci, B.; Hratchian, H. P.; Ortiz, J. V.; Izmaylov, A. F.; Sonnenberg, J. L.; Williams-Young, D.; Ding, F.; Lipparini, F.; Egidi, F.; Goings, J.; Peng, B.; Petrone, A.; T. Henderson; D. Ranasinghe; V. G. Zakrzewski; J. Gao; N. Rega; G. Zheng; W. Liang; M. Hada; M. Ehara; K. Toyota; R. Fukuda; J. Hasegawa; M. Ishida; T. Nakajima; Y. Honda; O. Kitao; H. Nakai; T. Vreven; K. Throssell; Montgomery, J., J. A. ; Peralta, J. E.; Ogliaro, F.; Bearpark, M. J.; Heyd, J. J.; Brothers, E. N.; Kudin, K. N.; Staroverov, V. N.; Keith, T. A.; Kobayashi, R.; Normand, J.; Raghavachari, K.; Rendell, A. P.; Burant, J. C.; Iyengar, S. S.; Tomasi, J.; Cossi, M.; Millam, J. M.; Klene, M.; Adamo, C.; Cammi, R.; Ochterski, J. W.; Martin, R. L.; Morokuma, K.; Farkas, O.; Foresman, J. B.; Fox, D. J. *Gaussian 16, Revision A.03*, Gaussian, Inc.: Wallingford CT, 2016.
4. Peng, C.; Ayala, P. Y.; Schlegel, H. B.; Frisch, M. J. *J. Comput. Chem.* **1996,** *17*, 49-56. doi:10.1002/(SICI)1096-987X(19960115)17:1<49::AID-JCC5>3.0.CO;2-0
5. Peng, C.; Schlegel, H. B. *Israel J. Chem.* **1993,** *33*, 449-454. doi:10.1002/ijch.199300051
